# Characteristics Influencing Support for the National Health Service COVID-19 App in England and Wales: Findings From a Longitudinal Survey

**DOI:** 10.2196/76863

**Published:** 2026-01-28

**Authors:** Josephine Exley, Paul Boadu, Kasim Allel, Bob Erens, Nicholas Mays, Mustafa Al-Haboubi

**Affiliations:** 1 London School of Hygiene & Tropical Medicine London United Kingdom; 2 Nuffield Department of Primary Care Health Sciences University of Oxford Oxford United Kingdom

**Keywords:** contact tracing apps, COVID-19, infection prevention and control, public health, test and trace

## Abstract

**Background:**

The use of proximity (contact) tracing mobile phone apps during the COVID-19 pandemic to support manual contact tracing was novel. Uptake of the app was lower than expected.

**Objective:**

We sought to identify distinct subgroups of individuals based on their level of support for the National Health Service (NHS) COVID-19 app in the first 15 months of the app’s implementation, and to identify the attitudes and characteristics associated with membership of more and less supportive groups.

**Methods:**

We conducted 8 waves of a longitudinal survey data of smartphone users, recruited from an online panel (n=2023 at baseline and n=1198 at survey wave 6) between October 14, 2020, and December 13, 2021. We used latent class analysis to identify subgroups of individuals with different inclinations of support for the NHS COVID-19 app. Sankey diagram analysis was used to assess individuals whose subgroup changed over the study period. We estimated population-weighted multinomial logistic regression models using sociodemographic characteristics as independent variables.

**Results:**

We identified 4 subgroups in survey waves 1 to 4—“not supportive” (1765/7210, 25%), “ambivalent” (2124/7210, 30%), “somewhat supportive” (1421/7219, 20%), and “completely supportive” (1900/7210, 26%). At wave 5, a total of 3 subgroups of support for the app emerged—“not supportive” (549/1613, 34%), “ambivalent” (497/1613, 31%), and “supportive” (567/1613, 35%). From wave 6 onward, the results showed 4 subgroups emerging—“least supportive” (1568/6952, 23%), “less supportive” (1179/6952, 17%), “ambivalent” (2105/6952, 30%), and “supportive” (2100/6952, 29%). The majority of respondents remained within their identified subgroups between survey waves. Among those who moved into different subgroups, most moved into a less supportive subgroup. Exceptions to this were from waves 2 to 3 and from waves 3 to 4, when higher percentages of respondents moved into more supportive subgroups. The biggest movement to less supportive subgroups occurred after wave 1 (October 2020), when 38% (2740/7210) of respondents moved into a less supportive subgroup. The biggest movement to more supportive subgroups, on the other hand, occurred after wave 2, when 22% (1586/7210) of respondents moved into more supportive subgroups. Over the course of the 8 waves, the percentage of respondents in supportive subgroups declined from 56% (3353/5988) to 29% (1737/5988). Key characteristics of more supportive individuals included having higher levels of trust in the government to control the spread of COVID-19 and having the app installed, while those less concerned about the risk COVID-19 posed to the country were more likely to be unsupportive (*P*<.05).

**Conclusions:**

When the app was launched, just over half of respondents were supportive, but this declined over the following 15 months. The attrition in support poses important challenges for governments to the use of apps in future pandemics. A potential reason was mistrust in the government’s handling of the pandemic.

## Introduction

As part of the response to the COVID-19 pandemic, several countries deployed novel proximity (contact) tracing mobile phone apps to supplement manual contact tracing efforts. They keep a temporary record of app users who have been in close contact with each other to alert them in case one user subsequently reports through the app contracting the virus. Evidence highlights the potential for apps to reduce the spread of COVID-19 [1-7]. For example, the National Health Service (NHS) COVID-19 app was estimated to have averted approximately 1 million cases and 10,000 deaths in England and Wales in its first year [8], and for every percentage increase in uptake the number of cases was estimated to have been reduced by up to 2% [9].

The effectiveness of such technology depends on the level of uptake among the population [1,10-12]. In settings where their use was not mandated, uptake was reliant on individuals’ willingness to install and use the app. However, uptake of contact tracing apps in many countries was found to be lower than anticipated [13-17], although surveys conducted before the introduction of apps suggested high levels of public support for the use of these apps as part of the pandemic response [18,19]. Evidence from our own work following a cohort of smartphone users in England and Wales during the first year of implementation of the NHS COVID-19 app found that despite changes in policy and case numbers, installation of the app remained relatively stable at around 50% [20]. The majority of those who had ever installed the app did so soon after the app’s launch.

With scientists predicting a growing probability of future pandemics [21], contact tracing apps are likely to become an increasingly ubiquitous public health tool. As such, there is a need to learn how to improve the implementation and uptake of such apps in the face of likely future pandemics. In this analysis of a longitudinal 8-wave survey of a representative sample of adult smartphone users in England and Wales, we aimed to identify distinct subgroups of individuals based on their level of support for the NHS COVID-19 app in the first 15 months of the app’s implementation and to identify the attitudes and characteristics associated with membership of more and less supportive groups.

## Methods

This study draws on longitudinal (prospective cohort) survey data of smartphone users aged 18 to 79 years in England and Wales. Details of the study have been described previously [20].

### Setting

The NHS COVID-19 app became part of the NHS Test and Trace Programme in July 2020 and was launched in England and Wales on September 24, 2020 (Textbox 1). It alerted close contacts of individuals who later tested positive for coronavirus to self-isolate. Additional features allowed users to check their symptoms, book a COVID-19 test, and check in at a venue. The survey started 2 weeks after the app was launched and coincided with rising COVID-19 cases and the tightening of COVID-19 restrictions. Multimedia Appendix 1 maps the 8 rounds of the online survey against the number of new COVID-19 cases reported and the key policy changes introduced by the governments in England and Wales during the study period. COVID-19 cases were highest during survey wave 3 (conducted between December 28, 2020, and January 6, 2021) and survey wave 8 (conducted between November 25, 2021, and December 13, 2021). Cases were lowest during survey wave 5 (conducted between March 15 and 31, 2021).

**Overview of the National Health Service (NHS) COVID-19 app and the key COVID-19 restrictions in place in England and Wales during the first year of the app’s launch.**

The NHS COVID-19 contact tracing app, using a decentralized model, was launched in England and Wales on September 24, 2020, for users aged 16 years and older and was made available in 12 languages.

It required a recent smartphone operating system and only collected users’ postcode areas to provide local risk information. By July 2022, it had over 31 million downloads [22]. The app sent anonymous alerts to close contacts (determined by proximity and duration algorithms) of users who reported positive COVID-19 tests in the app [23]. Unlike public health authorities’ self-isolation instructions (which ended in February 2022), app guidance was not legally binding. Users needed Bluetooth enabled for contact tracing, with an option within the app to temporarily disable it. Other features included symptom checking, test ordering, venue check-ins by QR codes (mandatory in England from September 2020 to February 2022 and a recommendation in Wales), and a self-isolation timer.

The app’s launch coincided with rising COVID-19 cases and the tightening of restrictions [24]. Wales introduced a 2-week firebreak on October 23, while England entered a second national lockdown on November 5. Both countries briefly returned to tiered restrictions in December before reimposing lockdowns by year-end. In early 2021, England entered a third lockdown, and Wales maintained the highest restriction level (“stay at home”). Restrictions were slowly eased throughout spring and summer 2021, with most lifted by July 19, including the need to self-isolate if double vaccinated. The emergence of the Omicron variant in November 2021 once again marked the tightening of restrictions.

As shown in Multimedia Appendix 1, survey wave 1 (October 14-22, 2020) took place after the introduction of the local 3-tier system of COVID-19 restrictions; at that time, it was a legal requirement for venues in England to take the contact details of visitors either manually or by scanning a QR code, while in Wales this was a recommendation only. Survey wave 2 (November 12-23, 2020) was conducted during the second national lockdown, introduced on November 5, and followed a 2-week firebreak in Wales from October 23 to November 8 in which the public were instructed to stay at home and the hospitality industry and nonessential restaurants had to close. Survey wave 3 (December 28, 2020, to January 6, 2021) was conducted over the Christmas period when the country had returned to the tier system; the southeast of England and the whole of Wales moved to a newly created tier 4 (“stay at home”), the first vaccine was administered in the United Kingdom on December 8, 2020, and England entered the third national lockdown on January 6, 2021. Survey wave 4 (February 1-15, 2021) occurred while England remained in the third national lockdown and Wales remained at tier 4. Survey wave 5 (March 15-31, 2021) took place at the start of the easing of restrictions, after the government in England outlined a 4-step plan; by the start of the survey, schools had reopened and outdoor mixing in groups of up to 6 was allowed from March 29. Survey wave 6 (July 1-18, 2021) occurred ahead of the final step of easing restrictions in both England and Wales, when indoor venues had reopened and the public were allowed to meet in groups of up to 30 outdoors and 6 indoors. Survey wave 7 (August 31-September 13, 2021) took place after all legal limits on social contact had been lifted, including the requirement to check in to venues in England, and those who had been double vaccinated were no longer required to self-isolate if they had come into contact with someone who tested positive for COVID-19, provided they did not have any symptoms. Survey wave 8 (November 25-December 13, 2021) occurred at the same time as the Omicron variant was first being reported; a total of 6 southern African countries were placed on the travel red list and the first cases were detected in the United Kingdom, and on December 8 the government announced that Plan B measures were to be reintroduced, including mandatory face coverings in most indoor settings, working from home, and use of the NHS COVID Pass for entry into nightclubs and settings where large crowds gather. Data on daily new cases were obtained from the UK Coronavirus Dashboard, and information on lockdown and restriction timelines from the Institute for Government’s coronavirus timeline.

### Participants

We were interested in looking at differences in the usage of the app between different sociodemographic groups, such as age, level of education, social grade, health status, and minoritized ethnic groups. We expected to find differences in attitudes between members of our sample in different social grades (nonmanual vs manual). In order to detect a 6% difference (at 80% power and a 95% significance level) in attitudes between these 2 subgroups, a sample size of 1657 is required. More importantly, we wanted to detect differences in app use over time. Assuming about two-thirds of the sample at baseline were using the app, we would be able to detect about a 3% change in the overall sample between baseline/wave 1 and survey wave 2 with 95% confidence.

A representative sample of smartphone users aged 18 to 79 years was recruited through YouGov’s volunteer online panel, with quotas set on age, gender, and social grade. Panel members are recruited from different sources, including through advertising and partnerships with a range of websites. Sociodemographic data are collected when they join the panel. Participants are invited from the panel in a way that seeks to generate a nationally-representative sample [25].

In total, 2023 participants were recruited at the start of the study (referred to as the “baseline sample”). Panel members who completed the baseline survey were invited to all follow-up waves. After the fifth round of data collection, we recruited an additional 1198 participants to increase the sample size (referred to as the “additional sample”). At survey wave 8, 61% (1233/2023) of the main sample and 71% (848/1198) of the additional sample responded. The response rate for each sample at each survey wave is provided in Table S1 in Multimedia Appendix 2.

### Data Collection and Variables of Interest

Online surveys were undertaken roughly every 6 weeks between October 14, 2020, and December 13, 2021 (Multimedia Appendix 1). The survey was developed by the study team and adapted from previous surveys. It included questions on attitudes toward the app, including the level of support for it (measured on a 5-point Likert scale from 1 “not at all supportive” to 5 “completely supportive”), as well use of the app, perceptions of COVID-19, governments’ response to the pandemic, and demographic information (ie, age, sex, ethnicity, income, employment, etc). The core content of the questionnaire remained the same across all waves, but some questions were added or removed in response to the evolving pandemic.

The survey was administered by YouGov and sent to respondents by email. Participants who completed the first survey (survey wave 1 for the baseline sample or wave 6 for the additional sample) were invited to participate in all subsequent waves of the survey.

### Analysis

#### Determining Latent Classes of Support for NHS COVID-19 App

We used latent class analysis (LCA) to identify distinct subgroups of individuals with similar levels of support for the app at each survey wave [26] using the *gsem* function in Stata [27]. We considered LCA an optimal strategy due to its robustness against potential measurement errors, such as potential biases introduced by the timing of the questionnaire, and its ability to parsimoniously model restricted latent groups across multiple levels of support. This approach also helps prevent measurement errors that may arise from assigning respondents to groups based on their survey responses without recourse to statistical justification. To determine the optimal number of classes to include in the LCA model at each survey wave, we estimated the Bayesian information criteria (BIC) and Akaike information criteria (AIC) maximum likelihood values, with lower values indicating a better fit for the number of subgroups [28,29]. The results were similar when the test was conducted with and without covariates, including general health, disability, vulnerability to COVID-19, region, household income, tenure, app installation, trust in government, age, ethnicity, and sex. The results of the maximum likelihood test are presented in Table S2 in Multimedia Appendix 2.

#### Sankey Diagram Analysis

To assess the movement of individuals between subgroups of support for the app over survey waves, we constructed a Sankey diagram of the wave-specific estimated classes and examined individual transitions between identified support subgroups over the survey period [30,31]. The analysis was restricted to individuals who responded to 2 consecutive waves.

#### Regression Analysis

To examine the factors associated with belonging to a given subgroup of support for the app, we estimated population-weighted multinomial logistic regression models at three timepoints: survey wave 1, survey wave 5, and survey wave 8. We opted to estimate the regression model at the study start and endpoints, but additionally included wave 5 as it represents a notable change in the distribution of LCA results, in that the number of subgroups reduced from 4 to 3. The definition of dependent and independent variables included in the multinomial logistic regression model are presented in Table S3 in Multimedia Appendix 2.

To select the independent variables for the model and mitigate multicollinearity, a correlation matrix (Tables S4-S6 in Multimedia Appendix 2) and variance inflation factors (VIF) (Tables S7-S9 in Multimedia Appendix 2) were generated. Based on the results, the following explanatory variables were included in the model: age, gender, ethnicity, self-reported health status, whether day-to-day activities were limited because of a health problem or disability, perceived vulnerability to COVID-19, region of residence, household income, household ownership, whether the app was installed at the current survey wave, whether the respondent had a COVID-19 infection since the previous survey, extent of trust in the government to control the spread of COVID-19, extent of concern about the risk COVID-19 poses to oneself, and extent of concern about the risk COVID-19 poses to the country. Fisher statistics are reported for measuring the model’s goodness-of-fit. All statistical analyses were performed using Stata Standard Edition (version 18; StataCorp LLC) software. The research is reported in line with the STROBE (Strengthening the Reporting of Observational Studies in Epidemiology) Statement for cohort studies [32] (Multimedia Appendix 3).

### Ethical Considerations

Consent was sought before starting the first survey but not at subsequent waves. Responses were anonymized by YouGov before being passed to the researchers. Participants were free to withdraw at any time without needing to provide a reason. Ethics approval was obtained from the London School of Hygiene & Tropical Medicine Research Ethics Committee (reference number 22483). Participants receive “points” for completing surveys, which can subsequently be converted into cash payments.

## Results

### Overview

Overall, a total of 3221 participants were recruited to the study (2023 at survey wave 1 and 1198 at survey wave 6). The characteristics of participants are presented in Table 1. About 53% (1118/2023) of participants at wave 1 and 53% (619/1198) of participants at wave 6 were female, 42% (810/2023) and 39% (467/1198) were aged 40 years or younger, 86% (2775/2023) and 87% (1045/1198) were White, and 61% (1235/2023) and 57% (679/1198) had received higher education in the baseline and additional samples, respectively.

**Table 1:** Characteristics of respondents to an 8-wave longitudinal survey on use and views on the National Health Service (NHS) COVID-19 app (October 2020 to December 2021). A currency exchange rate of GBP £1=US $1.32 is applicable.

Characteristics		Baseline sample (n=2023), n (%)	Additional sample (n=1198), n (%)
**Sex**			
	Male	905 (47.2)	579 (48.3)
	Female	1118 (52.8)	619 (51.7)
**Age group (years)**			
	18-29	439 (23.1)	218 (18.2)
	30-39	371 (18.4)	249 (20.8)
	40-49	381 (20.8)	220 (18.4)
	50-59	381 (17.5)	210 (17.5)
	60-69	298 (12.1)	192 (16.0)
	70-79	153 (8.1)	109 (9.1)
**Ethnicity**			
	White	1775 (86.1)	1045 (87.2)
	All other ethnic groups	248 (13.9)	153 (12.8)
**Region**			
	North East	103 (4.4)	30 (2.5)
	North West	252 (12.4)	102 (8.5)
	Yorkshire and the Humber	185 (9.1)	94 (7.8)
	East Midlands	174 (8.0)	74 (6.2)
	West Midlands	176 (9.9)	63 (5.3)
	East of England	209 (10.4)	84 (7.0)
	London	283 (15.2)	163 (13.6)
	South East	320 (15.8)	101 (8.4)
	South West	197 (9.5)	69 (5.8)
	Wales	124 (5.4)	418 (34.9)
**Highest level of education attainment**			
	No formal qualifications	78 (3.5)	42 (3.5)
	GCSE^a^ or equivalent	232 (11.3)	143 (11.9)
	A-level or equivalent	293 (15.6)	200 (16.7)
	Higher education	1235 (60.8)	679 (56.7)
	Other	137 (6.4)	92 (7.7)
	Prefer not to say or did not answer	48 (2.5)	42 (3.5)
**Employment status**			
	Currently working	1151 (56.8)	693 (57.9)
	Not currently working	92 (4.7)	33 (2.8)
	Voluntary work or carer	96 (4.6)	58 (4.8)
	Unemployed or permanent sick leave	222 (11.0)	102 (8.5)
	Education	81 (5.7)	80 (6.7)
	Retired	344 (15.6)	215 (18.0)
	Other	37 (1.7)	17 (1.4)
**Household income (£)**			
	<14,999	224 (10.8)	130 (10.9)
	15,000-24,999	285 (14.0)	197 (16.4)
	25,000-34,999	251 (12.7)	154 (12.9)
	35,000-60,000	431 (20.9)	227 (19.0)
	>60,000	346 (17.5)	188 (15.7)
	Prefer not to say or did not answer	486 (24.1)	302 (25.2)
**Personal income (£)**			
	<14,999	510 (25.7)	300 (25.0)
	15,000-24,999	382 (18.4)	237 (19.8)
	25,000-34,999	319 (15.6)	157 (13.1)
	35,000-60,000	267 (12.7)	154 (12.9)
	>60,000	107 (5.5)	43 (3.6)
	Prefer not to say or did not answer	438 (22.0)	307 (25.6)
**IMD^b^**			
	1–most deprived	351 (17.9)	218 (18.2)
	2	373 (18.0)	246 (20.6)
	3	408 (20.3)	233 (19.5)
	4	442 (21.3)	231 (19.3)
	5–least deprived	448 (22.5)	269 (22.5)
**Living arrangements**			
	lives alone	372 (18.3)	211 (17.6)
	other adult(s), no children	1151 (55.4)	633 (52.8)
	children, no other adults	49 (2.4)	32 (2.7)
	other adult(s) and children	451 (23.9)	322 (26.9)
**Household ownership**			
	Own	1169 (55.9)	683 (60.1)
	Rent	589 (29.5)	292 (25.7)
	Live with friends or family	233 (13.0)	133 (11.7)
	Other	32 (1.6)	28 (2.5)
**Self-reported health status**			
	Very good	535 (27.1)	297 (24.8)
	Good	988 (49.2)	572 (47.8)
	Fair	352 (16.6)	234 (19.5)
	Bad or very bad	117 (5.4)	72 (6.0)
	Not answered	31 (1.7)	23 (1.9)
**Day-to-day activities limited because of a health problem or disability lasting (or expected to last) at least 12 months**			
	Limited a lot	159 (7.3)	108 (9.0)
	Limited a little	325 (15.5)	195 (16.3)
	No	1498 (74.9)	863 (72.0)
	Not answered	41 (2.2)	32 (2.7)

^a^GCSE: General Certificate of Secondary Education.

^b^IMD: index of multiple deprivation based on respondent’s usual place of residence [33].

### Latent Classes of Support for the App

The test results (BIC/AIC) of the number of latent classes show 4 subgroups of population with differences in support for the NHS COVID-19 app for all survey waves, except for wave 5, where 3 subgroups were identified (Table S2 in Multimedia Appendix 2). The distribution of the identified subgroups in each survey wave is presented in Table 2.

**Table 2:** Distribution of subgroups of support for the National Health Service (NHS) COVID-19 app across 8 survey waves (W1–W8) from an 8-wave longitudinal survey conducted between October 2020 and December 2021 (n=number of observations per wave; W=survey wave).

Level of support	W1 (Oct 14-22, 2020; n=2023), %	W2 (Nov 12-23, 2020; n=1781), %	W3 (Dec 28, 2020-Jan 6, 2021; n=1732), %	W4 (Feb 1-15, 2021; n=1674), %	W5 (Mar 15-31, 2021; n=1613), %	W6 (Jul 1-18, 2021; n=2667), %	W7 (Aug 31-Sep 13, 2021; n=2204), %	W8 (Nov 25-Dec 13, 2021; n=2081), %
Least supportive	16^a^	28^a^	28^a^	27^a^	25^a^	20	23	25
Less supportive	—	—	—	—	—	17	18	16
Ambivalent	28	32	29	29	36	32	29	30
Somewhat supportive	20	19	20	19	39^b^	32^b^	29^b^	29^b^
Completely supportive	36	21	22	25	—	—	—	—

^b^This percentage value applies to both the “least supportive” and “less supportive” subgroups; in Waves 1-5 these categories were statistically measured as a single latent class.

^b^This percentage value applies to both the “somewhat supportive” and “completely supportive” subgroups; in Waves 5-8 these categories were statistically measured as a single latent class.

ᵃThis percentage value applies to both the “least supportive” and “less supportive” subgroups; in Waves 1-5 these categories were measured as a single latent class.

The characteristic of individuals belonging to the identified subgroups were similar for survey waves 1-4 and survey waves 6-8, so they were presented and analyzed together.

From survey waves 1 to 4 (October 14, 2020, to February 15, 2021), we identified 4 subgroups of support for the app. Table 3 shows the characteristics of the 4 population subgroups with different levels of support for the NHS COVID-19 app. Individuals in subgroup 1 were either not supportive at all or least supportive of the app, with good health and no disability, not vulnerable to COVID-19, generally equally distributed across all income groups and regions, and the majority were homeowners. Most had never installed the app and had no trust in the government to control the spread of COVID-19. The average age of individuals in subgroup 1 was 46 years, and they comprised similar proportions of individuals from all ethnic groups, with higher proportions of males. Based on these characteristics, we labeled this population subgroup as “not supportive.”

**Table 3:** Summary of characteristics of individuals belonging to the 4 subgroups of support for the National Health Service (NHS) COVID-19 app (survey wave 1 to wave 4 [October 14, 2020, to February 15, 2021]). Table S10 in Multimedia Appendix 2 provides underlying statistics.

Characteristic	Subgroup 1: Not Supportive	Subgroup 2: Ambivalent	Subgroup 3: Somewhat Supportive	Subgroup 4: Completely Supportive
Support	Not at all supportive, 51% (901/1765) or least supportive, 49% (863/1765)	Indifferent about support for the app, 100% (2124/2124)	Somewhat supportive, 100% (1421/1421)	Completely supportive of the app, 100% (1900/1900)
General health	Majority in good health, 52% (813/1765)	Majority in good health, 53% (1134/2124)	Majority in good health, 53% (754/1421)	Higher proportion with good health, 46% (871/1900)
Disability	Majority without disability, 75% (1317/1765)	Majority without disability, 75% (1598/2124)	Majority without disability, 75% (1060/1421)	Majority without disability, 72% (1359/1900)
Vulnerability to COVID-19	Majority not vulnerable, 59% (1039/1765)	Majority not vulnerable, 59% (1258/2124)	Majority not vulnerable, 57% (815/1421)	Majority vulnerable, 51% (960/1900)
Region	Generally evenly spread across all regions	Generally evenly spread across all regions	Generally evenly spread across all regions	Generally evenly spread across all regions
Household income	Generally evenly spread across income groups	Generally evenly spread across income groups	Generally evenly spread across income groups	Generally evenly spread across income groups
Tenure	Majority homeowners, 58% (1015/1765)	Majority homeowners, 56% (1181/2124)	Majority homeowners, 58% (831/1421)	Majority homeowners, 64% (1213/1900)
App installation	Majority never installed, 62% (1093/1765)	Higher proportion never installed, 44% (935/2124)	Majority installed, 59% (845/1421)	Majority installed, 74% (1409/1900)
Trust	Higher proportion with not at all trust, 84% (854/1765)	Higher proportion with little trust, 38% (803/2124)	Higher proportion with little trust, 37% (521/1421)	Higher proportion with fair amount of trust, 34% (646/1900)
Age (average)	46 years	45 years	45 years	49 years
Ethnicity	Similar proportion across all ethnic groups	Higher proportions among other ethnic groups, 35% (298/855)	Similar proportion across all ethnic groups	Higher proportions among White individuals, 27% (1714/6355)
Sex	Higher proportion of males, 27% (859/3232)	Higher proportion of females, 33% (1281/3978)	Similar proportions of males and females	Higher proportion of males, 28% (890/3232)

Most of the individuals in subgroup 2 neither supported nor opposed the NHS COVID-19 app and indicated that they had good health and were not vulnerable to COVID-19. Most were homeowners, had never installed the app, and did not have very much trust in the government to control the spread of COVID-19. Most were from ethnic groups other than White, with a higher proportion being female, and their average age was 45 years.

They were equally distributed across regions and household incomes, with a higher proportion being female. We labeled this subgroup “Ambivalent” based on their characteristics.

The third subgroup was labeled “Somewhat supportive” because individuals in this group had some level of support for the app. Most were in good health, had no disability, and did not consider themselves vulnerable to COVID-19. A higher proportion had never installed the app and did not have much trust in the government to control the spread of COVID-19. Individuals in this subgroup were generally equally distributed across regions, income groups, sex, and ethnic groups, and the majority were homeowners, with an average age of 45 years.

Individuals in the fourth subgroup were completely supportive of the NHS COVID-19 app, a higher proportion were in good health, and the majority had no disability. However, the majority also indicated they were vulnerable to COVID-19, were homeowners, had installed the app, and had a fair amount of trust in the government to control the spread of COVID-19. Similar to the other subgroups, individuals in this subgroup were generally equally distributed across all income groups and regions. A higher proportion of individuals in this group were of White ethnic group and were male. The average age of this subgroup was 49 years.

The number of subgroups identified in wave 5 (March 15-31, 2021) was reduced by one to 3. Table 4 shows the characteristics of individuals in each of the identified subgroups.

**Table 4:** Summary of characteristics of individuals belonging to the 3 subgroups of support for the National Health Service (NHS) COVID-19 app (survey wave 5 [March 15-31, 2021]). Table S11 in Multimedia Appendix 2 provides underlying statistics.

Characteristic	Subgroup 1: Not Supportive	Subgroup 2: Ambivalent	Subgroup 3: Supportive
Support	Not at all supportive, 52% (288/549) or least supportive, 48% (261/549)	Indifferent about support for app 100% (497/497)	Somewhat supportive, 46% (258/567) or completely supportive of the app, 54% (309/567)
App installation	Majority never installed, 56% (308/549)	Higher proportion either installed, 41% (205/497) or never uninstalled, 37% (186/497)	Majority installed, 73% (413/567)
Trust	Higher proportion with no trust at all, 35% (193/549)	Higher proportion with a fair amount of trust, 41% (202/497)	Higher proportion with a fair amount of trust, 42% (236/567)
Age (average)	47 years	45 years	48 years
Sex	Higher proportion of males, 37% (268/721)	Higher proportion of females, 34% (306/892)	Higher proportion of males, 36% (262/721)

Unlike the subgroups identified from study waves 1-4 (October 14, 2020, to February 15, 2021), characteristics such as general health, disability status, vulnerability to COVID-19, region, household income, and tenure were not statistically different among the 3 identified subgroups in wave 5. Based on the characteristics of individuals in subgroup 1, we labeled it “Not supportive.” Those in this subgroup were not at all or least supportive of the NHS COVID-19 app, the majority had never installed the app, and a higher proportion had no trust at all in the government to control the spread of COVID-19. The average age of this subgroup was 47 years, and a higher proportion were male. Following a similar approach, subgroups 2 and 3 were labeled “Ambivalent” and “Supportive,” respectively.

Furthermore, the results from survey waves 6-8 (July 1, 2021, to December 13, 2021) show the reemergence of 4 subgroups of support for the NHS COVID-19 app, with some subgroups showing different characteristics than those identified in survey waves 1-4 (Table 5). Based on the characteristics of the individuals in the identified subgroups, we labeled subgroups 1, 2, 3, and 4 as “Least supportive,” “Less supportive,” “Ambivalent,” and “Supportive,” respectively (Table 5).

**Table 5:** Summary of characteristics of individuals belonging to the 4 subgroups of support for the National Health Service (NHS) COVID-19 app (survey waves 6-8 [July 1, 2021, to December 13, 2021]). Table S12 in Multimedia Appendix 2 provides underlying statistics.

Characteristic	Least Supportive	Less Supportive	Ambivalent	Supportive
Support	Not at all supportive, 100% (1568/1568)	Least supportive, 100% (1179/1179)	Indifferent about support for app, 100% (2105/2105)	Somewhat supportive, 48% (999/2100) or completely supportive of app, 52% (1101/2100)
General health	Higher proportion in good health, 48% (745/1568)	Higher proportion in good health, 51% (607/1179)	Higher proportion in good health, 50% (1043/2105)	Higher proportion in good health, 48% (1016/2100)
Disability	Majority without disability, 71% (1109/1568)	Majority without disability, 77% (903/1179)	Majority without disability, 70% (1484/2105)	Majority without disability, 69% (1444/2100)
Region	Higher proportion in Wales, 24% (318/1301)	Higher proportions in London and South and the North	Higher proportion in Midlands and East of England	Higher proportion in London and South and Wales
Household income	Generally evenly spread across income groups	Generally evenly spread across income groups	Generally evenly spread across income groups	Generally evenly spread across income groups
Tenure	Majority homeowners, 64% (1008/1568)	Majority homeowners, 61% (721/1179)	Majority homeowners, 59% (1246/2105)	Majority homeowners, 64% (1335/2100)
App installation	Majority never installed, 68% (1074/1568)	Higher proportion never installed, 47% (549/1179)	Higher proportion installed, 43% (901/2105)	Majority installed, 76% (1604/2100)
Trust	Higher proportion with no trust at all, 40% (630/1568)	Higher proportion with little trust, 37% (436/1179)	Higher proportion with a fair amount of trust, 37% (781/2105)	Higher proportion with a fair amount of trust, 40% (834/2100)
Age (average)	49 years	46 years	48 years	49 years
Sex	Higher proportion of males, 26% (856/3289)	Higher proportion of females, 18% (675/3663)	Higher proportion of females, 33% (1216/2105)	Higher proportion of males, 32% (1040/3289)

The Sankey diagram presented in Multimedia Appendix 4 shows the movement of respondents between classes over the course of the surveys. Between each survey wave, more than half of respondents did not change class. Among those who did, most moved into a less supportive class (measured by the thickness of the palette), except between survey waves 2 to 3 and 3 to 4, where more individuals moved upwards into more supportive classes than downwards. The biggest movement downwards occurred after survey wave 1 (October 2020) when 38% (275/723) of individuals moved into a less supportive class, half of whom (130/723, 18%) moved out of the completely supportive class to the somewhat supportive class, and over a third (108/723, 15%) moved into the Ambivalent subgroup. The biggest movement upwards occurred after survey wave 2 (November 2020) with 22% (233/1060) of individuals moving to more supportive classes. As a result of the greater number moving into less supportive classes, the percentage of individuals in “supportive” subgroups decreased over time, from 56% (1131/2023; “completely” or “somewhat supportive”) at survey wave 1 to 29% (611/2081; “supportive”) by survey wave 8.

### Factors Associated With Class Membership

To quantify the factors associated with the likelihood of an individual’s membership in a particular subgroup at survey waves 1 (October 2020), 5 (March 2021), and 8 (December 2021), we calculated the relative risk ratio (RRR) of variables being present in a particular subgroup relative to the reference group (completely supportive). An RRR<1 suggests a lower likelihood relative to the reference group, while an RRR>1 indicates a higher likelihood of the variable’s presence. The full results of the multinomial logistic regression are presented in Tables S13-S15 in Multimedia Appendix 2 and summarized in Multimedia Appendix 5.

At all 3 waves, individuals who never had the app installed and had less trust in government to control the spread of COVID-19 were associated with a relative reduction in the likelihood of being in the most supportive subgroups compared with all other subgroups. For example, never having the app installed reduced the likelihood of being in the most supportive group compared with the least supportive group (RRR 0.03, 95% CI 0.02-0.05 at survey wave 1; RRR 0.07, 95% CI 0.06-0.10 at survey wave 5; and RRR 0.03, 95% CI 0.02-0.05 at survey wave 8). Similarly, having little or no trust in the government to control the spread of COVID-19 reduced the likelihood of being in the most supportive group compared with the least supportive group (RRR 0.19, 95% CI 0.12-0.29 at survey wave 1; RRR 0.27, 95% CI 0.20-0.38 at survey wave 5; and RRR 0.30, 95% CI 0.19-0.47 at survey wave 8).

At all 3 waves, not being at all concerned about the risks COVID-19 posed to the country was associated with a reduced likelihood of being in the most supportive subgroups compared with the least supportive subgroup. Compared with individuals who were very concerned about the risks COVID-19 posed to the country, being not at all concerned increased the likelihood of being in the “not supportive” group at survey wave 1 (RRR 10.87, 95% CI 2.63-44.85) and survey wave 5 (RRR 9.92, 95% CI 3.03-32.48) and increased the likelihood of being in the “least supportive” subgroup at survey wave 8 (RRR 13.72, 95% CI 2.56-73.62).

## Discussion

### Overview

We examined how levels of support for the COVID-19 app changed in the 15 months following its launch in England and Wales. We found just over half of respondents were supportive of the app around the time of its launch and that support declined over time, with a notable drop-off in those who were completely supportive occurring between the first (October 14-22, 2020) and second (November 12-23, 2020) survey wave. From survey wave 6 (July 1-18, 2021), more respondents were unsupportive than supportive of the app. Key characteristics of individuals who were more supportive included having higher levels of trust in the government to control the spread of COVID-19, having the app installed, and being more concerned about the risk COVID-19 posed to the country.

The level of support observed in this study was lower than might have been expected based on studies conducted before the app was launched [18,19]. A contact tracing app was initially framed as a central component of the government’s strategy to control the spread of COVID-19, and media coverage in early 2020 was positive [34,35]. Yet following technical issues during piloting, including abandoning the first design [36], the government’s framing of the app changed from a technological solution to a more experimental technology [35]. The development process received prominent critical media coverage, which often presented contact tracing apps as controversial [37] and narrowed the debate to privacy concerns around what access authorities would have to personal data on users’ phones [38,39]. Among the cohort included in this study, being worried about privacy was the key reason for not downloading the app [20].

Lower levels of support by the time of the national launch also likely reflect wider public perceptions of the government’s handling of the pandemic. By autumn 2020, trust in the government to handle the pandemic was low [40,41], and its launch coincided with rising COVID-19 cases and the tightening of restrictions (Multimedia Appendix 1). Soon after the app’s launch, a range of new restrictions were introduced, including a second national lockdown and a modified tiered system [42]. The government’s approach was widely characterized as confusing and chaotic [43,44], exemplified by the last-minute scrapping of plans in England to relax restrictions over the 2020 Christmas period, and likely contributed to negative attitudes toward the government’s handling of the pandemic. In turn, this may have contributed to the perception that the app was ineffective at controlling the spread of COVID-19 and did not live up to earlier positive expectations [45,46].

Unlike levels of trust, which were found to fluctuate over the study period [41], levels of support consistently declined. Following the app’s launch, individuals’ mobility and social contact were highly restricted, with nonessential indoor leisure facilities and outdoor hospitality only reopening in April 2021. This might have fed perceptions that the app had limited utility at an individual level. Evidence on uptake indicates that the venue check-in feature was a driver of uptake [20]. Yet by July 2021, most social distancing requirements (including the requirement to check in to venues) were dropped. The shift observed at survey wave 6 (July 1-18, 2021), with more people reporting they were unsupportive than supportive for the first time, potentially indicates that individuals were no longer placing much value on the app. Among those who uninstalled the app, not finding it useful was a key reason for doing so [20]. When restrictions were introduced again in autumn 2021 in response to the Omicron variant, the requirement to check in to venues was not reintroduced, which potentially reinforced perceptions that the app did not have a major role to play in controlling the spread of COVID-19.

Declining support might also reflect perceptions around the reliability of the app. Issues experienced by users in the weeks following the app’s launch, including “ghost notifications,” were widely reported in the mainstream media [47], and analysis of reviews posted in app stores showed that problems associated with the app’s functionality were a key driver of negative comments [48,49]. As England and Wales approached so-called “freedom day” in July 2021, there was an increase in app users being told to self-isolate, coined the “pingdemic” in the media, and often reported as a shortcoming of the app rather than the direct result of a spike in infections due to the newly circulating Delta variant [50]. In response, the government decreased the sensitivity of the app [51]. This decision was criticized by the Opposition [52], and it likely reinforced the (incorrectly held) perception that the app was not working effectively.

Individuals who used the app were found to be more supportive throughout the study period. Based on our findings, it is not possible to determine whether being more supportive was a reason for installing the app or whether using the app increased support, though the fact that most people installed the app for the first time around the app’s launch in October 2020 suggests that the main direction of effect is likely from support in principle to take up the app. Since individuals who were more concerned about the risk COVID-19 posed to the country were more supportive of the app [53], these individuals potentially prioritized steps to control the spread of the virus over other concerns [54]. Evidence from elsewhere indicates that individuals who were more concerned about the risk of COVID-19 were more likely to engage in protective behaviors, including app use [20,55,56].

### Strengths and Limitations

The strength of this study is that it includes 8 waves of data collected over a 15-month period after the launch of the NHS COVID-19 app. At each timepoint, participants were asked about their level of support for the app, allowing us to examine changing trends in support.

The sample was representative of the general population of smartphone users in terms of age (up to 79 years), gender, social grade, and region. The sample were younger and more highly educated than the general population. This partly reflects smartphone ownership, which tends to be higher among younger individuals and those from higher-income households [57], but is also a reflection of the population that participates in nonprobability online panels [58]. One particular weakness of the sample is the underrepresentation of some minoritized groups. This is particularly important in light of evidence that some groups at higher risk of contracting COVID-19 were less likely to use the app [20].

The attrition rate was relatively high, with 61% of the original sample responding at survey wave 8. Nevertheless, we recruited new panel members ahead of survey wave 6 to top up the sample.

The question on support for the app could have been interpreted in different ways, and the results likely capture respondents’ support in principle for apps to control the spread of COVID-19 as well as specific support for the app as deployed. Using mixed methods that incorporate qualitative components would enhance surveillance systems by providing deeper insights into the public’s perceptions of contact tracing apps and which aspects of the app people are more or less supportive of.

### Implications

Evidence demonstrates that apps had a positive impact on controlling the spread of COVID-19 [8,9]. However, uptake of the app was disappointing compared to expectations based on reports from the start of the pandemic, and by the time the app launched, only 36% of our sample were completely supportive. As with many other countries, the deployment of a contact tracing app was novel in England and Wales. As such, some of the teething problems experienced during the development of the app are unlikely to be repeated in the future. However, while most countries, including England and Wales, have ended their COVID-19 contact tracing programs, it is likely that apps will continue to be included in future pandemic preparedness plans.

The results presented in this study provide important lessons and potential strategies as to how the governments in England and Wales might increase the impact of apps in the future. First, there is a need to develop a communication strategy that considers the different phases from app development through deployment and maintenance. In the future, when relying on untested technology, there is a need to be open and transparent during the development phase about the potential risks and benefits [45], avoiding overly optimistic messaging before the technology is proven. To improve public perceptions of effectiveness, an initial step would be to publicize the growing body of evidence that apps made a positive contribution to reducing transmission in the COVID-19 pandemic and be very clear from the outset about the functionality of the app, including what data the app collects and who these are shared with [59]. Given support was strongly influenced by trust in government, messaging might be better delivered by individuals and organizations in whom the public places greater trust [45,60]. More widely, support for an app is likely to depend on the degree to which the government of the day is trusted to be doing a reasonable job in responding to any pandemic or public health emergency.

There is a need to understand the optimal timing for launching an app to increase public perceptions that apps are an effective strategy to control the spread of COVID-19 at the population level, while also benefiting individuals in safely managing their daily lives. Given the wider contextual factors in autumn 2020, the launch of the app may have been mistimed. It might have been easier for the public to understand its benefit and utility if it had it been introduced as part of the government’s strategy to exit the lockdown between March and July 2021. Future research should examine how similar apps could be adapted to improve user experience and satisfaction [48].

### Conclusions

In this study among smartphone users in England and Wales, we found that the level of support for the NHS COVID-19 app was highest at launch and declined over the 15 months after rollout. A potential reason for the low and declining level of support was mistrust in both the Welsh and Westminster government’s handling of the pandemic, which likely contributed to a lack of support for the technology. The attrition in levels of support observed poses important challenges for the use of apps in future pandemics. However, our findings also show that individuals who installed the app and were more concerned about the risk of COVID-19 to the country were more supportive, suggesting that there is room to build support for apps especially among those concerned about the potential harm of a pandemic to others.

## Data Availability

The datasets generated or analyzed during this study are available on reasonable request. Requests should be directed to PIRU-pm@lshtm.ac.uk.

## Appendix

Multimedia Appendix 1 Overview of number of daily new cases of COVID-19 and key changes in policy measures in England and Wales (September 2020 to December 2021) [61,62].

Multimedia Appendix 2 Additional tables.

Multimedia Appendix 3 STROBE checklist.

Multimedia Appendix 4 Sankey diagram of respondents’ movement between subgroups over the course of 8 waves of a longitudinal survey on use and views of the National Health Service (NHS) COVID-19 app (October 2020 to December 2021), with n=1781 from wave 1 to wave 2, n=1612 from wave 2 to wave 3, n=1584 from wave 3 to wave 4, n=1509 from wave 4 to wave 5, n=1353 from wave 5 to wave 6, n=1249 from wave 6 to wave 7, and n=1124 from wave 7 to wave 8.

Multimedia Appendix 5 Summary of multinomial logistic regression model.

## Abbreviations

AIC: Akaike information criteria
BIC: Bayesian information criteria
LCA: latent class analysis
NHS: National Health Service
RRR: relative risk ratio
STROBE: Strengthening the Reporting of Observational Studies in Epidemiology
VIF: variance inflation factors
