# Supplementary figures and images for: Characteristics Influencing Support for the National Health Service COVID-19 App in England and Wales: Findings From a Longitudinal Survey
Source: J Med Internet Res. 2026 Jan 28;28:e76863. doi: 10.2196/76863 (PMC12895152; doi:10.2196/76863)

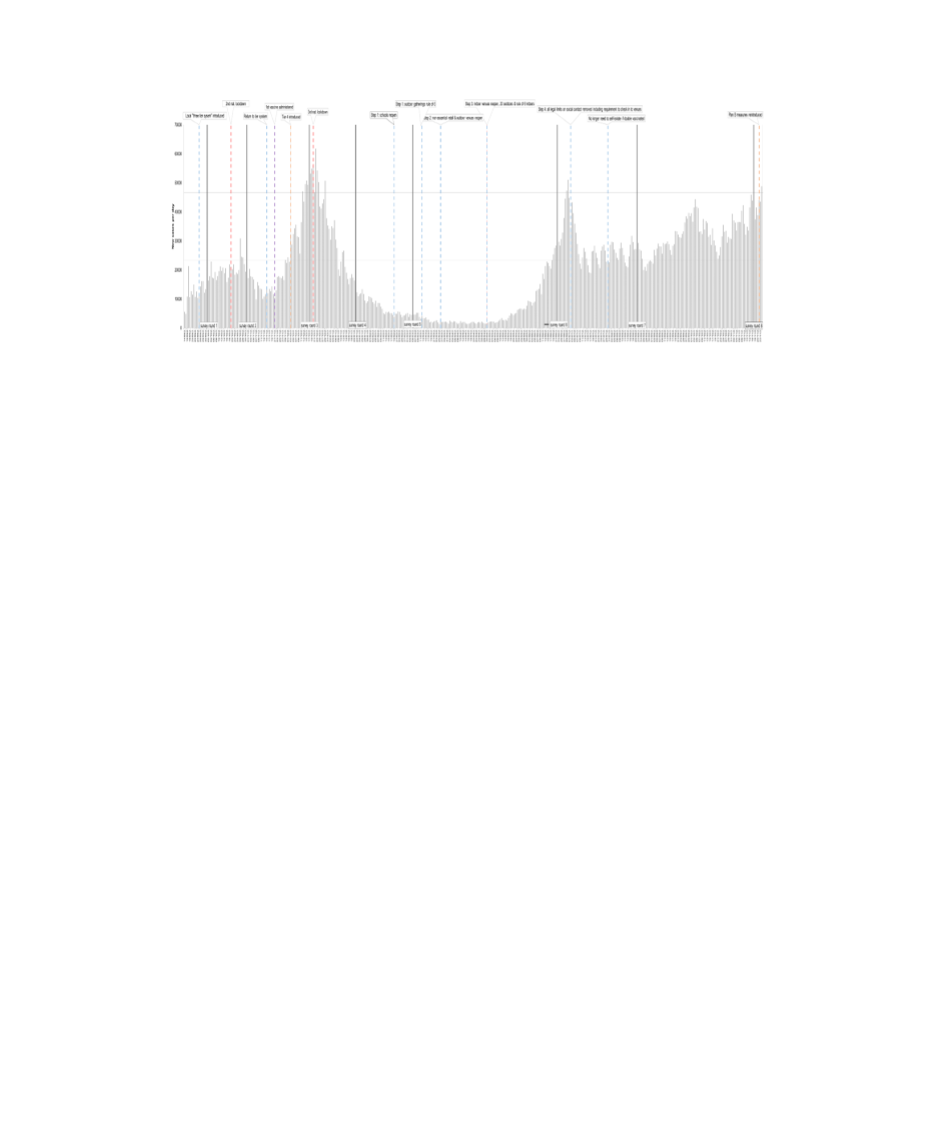

Supplement: Multimedia Appendix 1 [file jmir_v28i1e76863_app1.png]

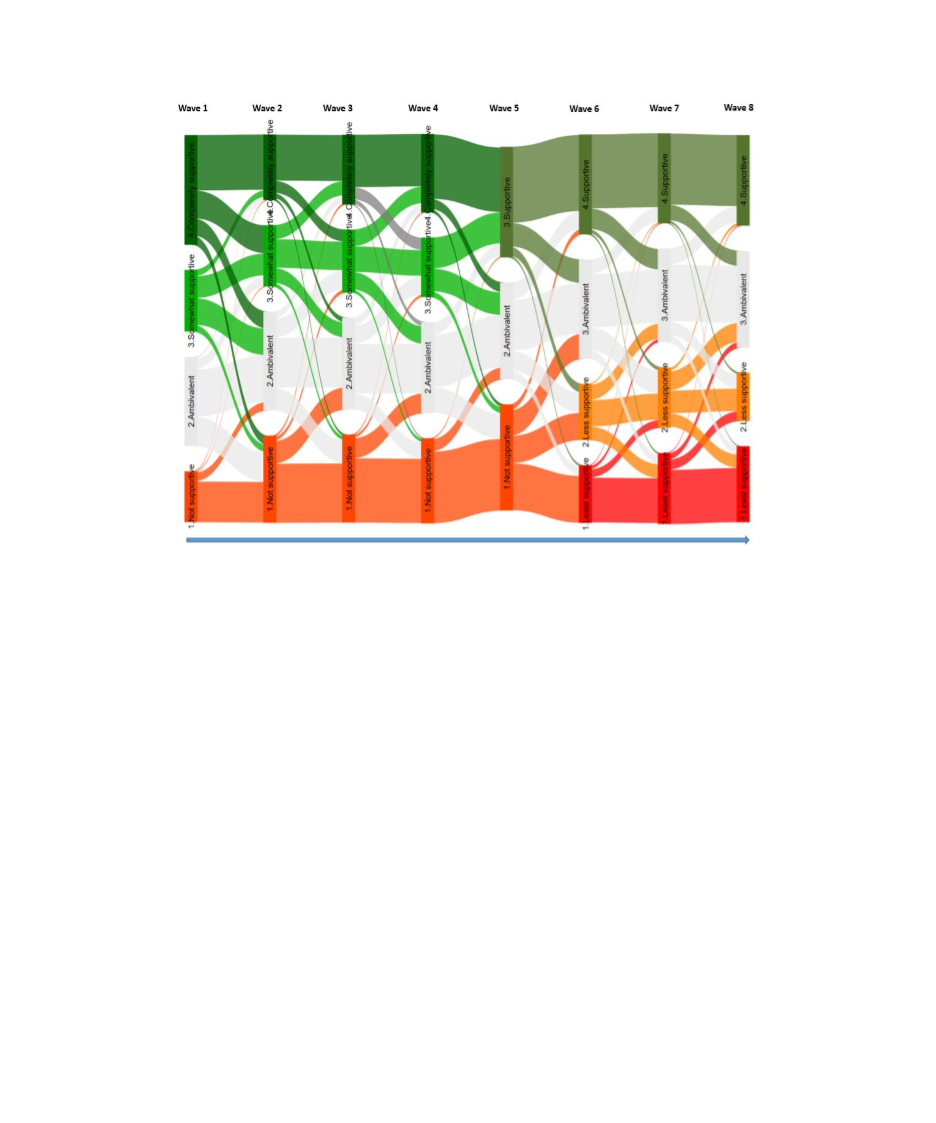

Supplement: Multimedia Appendix 4 [file jmir_v28i1e76863_app4.png]
