# Supplementary material for: Characteristics Influencing Support for the National Health Service COVID-19 App in England and Wales: Findings From a Longitudinal Survey
Source: J Med Internet Res. 2026 Jan 28;28:e76863. doi: 10.2196/76863 (PMC12895152; doi:10.2196/76863)
Supplement: Multimedia Appendix 2 [file jmir_v28i1e76863_app2.docx]

# Table S1: Response rate to a longitudinal survey on use and views on the NHS COVID-19 app

|  | **Baseline sample** | | **Additional sample** | |
| --- | --- | --- | --- | --- |
|  | **N** | **Response rate (%)** | **N** | **Response rate (%)** |
| w1: 14-22 Oct | 2,023 | 100.0 | - | - |
| w2: 12-23 Nov | 1,781 | 88.0 | - | - |
| w3: 28 Dec- 6 Jan | 1,732 | 85.6 | - | - |
| w4: 1-15 Feb | 1,674 | 82.7 | - | - |
| w5: 15-31 March | 1,613 | 79.7 | - | - |
| w6: 1-18 July | 1,469 | 72.6 | 1,198 | 100.0 |
| w7: 31 Aug – 13 Sep | 1,323 | 65.4 | 881 | 73.5 |
| w8: 25 Nov – 13 Dec | 1,233 | 60.9 | 848 | 70.8 |

# Table S2: Maximum likelihood test for number of latent subgroups

| **Model** | **log likelihood(model)** | **df** | **AIC** | **BIC** |
| --- | --- | --- | --- | --- |
| *WAVE 1 (n=2,023)* |  |  |  |  |
| class1 | -3337.83 | 2 | 6679.67 | 6690.89 |
| class2 | -3240.07 | 4 | 6488.14 | 6510.59 |
| class3 | -2955.75 | 6 | 5923.50 | 5957.18 |
| class4 | -2326.26 | 8 | 4668.53 | 4713.43 |
| class5 | -2326.26 | 10 | 4672.53 | 4728.65 |
| *WAVE 2 (n=1,781)* |  |  |  |  |
| class1 | -2988.86 | 2 | 5981.73 | 5992.70 |
| class2 | -2944.29 | 4 | 5896.58 | 5918.52 |
| class3 | -2794.01 | 6 | 5600.03 | 5632.94 |
| class4 | -2582.91 | 8 | 5181.82 | 5225.70 |
| *WAVE 3 (n=1,732)* |  |  |  |  |
| class1 | -2959.27 | 2 | 5922.54 | 5933.46 |
| class2 | -2893.46 | 4 | 5794.92 | 5816.75 |
| class3 | -2737.17 | 6 | 5486.33 | 5519.08 |
| class4 | -2533.10 | 8 | 5082.19 | 5125.85 |
| *WAVE 4 (n=1,674)* |  |  |  |  |
| class1 | -2864.36 | 2 | 5732.71 | 5743.56 |
| class2 | -2799.00 | 4 | 5606.00 | 5627.69 |
| class3 | -2637.18 | 6 | 5286.35 | 5318.89 |
| class4 | -2428.40 | 8 | 4872.80 | 4916.19 |
| *WAVE 5 (n=1,613)* |  |  |  |  |
| class1 | -2763.54 | 2 | 5531.07 | 5541.84 |
| class2 | -2714.20 | 4 | 5436.41 | 5457.95 |
| class3 | -2556.80 | 6 | 5125.61 | 5157.92 |
| class4 | -2556.80 | 8 | 5129.61 | 5172.70 |
| *WAVE 6 (n=2,667)* |  |  |  |  |
| class1 | -4542.28 | 2 | 9088.56 | 9100.34 |
| class2 | -4470.56 | 4 | 8949.12 | 8972.68 |
| class3 | -4209.28 | 6 | 8430.56 | 8465.89 |
| class4 | -3998.26 | 8 | 8012.52 | 8059.63 |
| *WAVE 7 (n=2,204)* |  |  |  |  |
| class1 | -3766.99 | 2 | 7537.98 | 7549.37 |
| class2 | -3687.10 | 4 | 7382.20 | 7405.00 |
| class3 | -3484.37 | 6 | 6980.75 | 7014.94 |
| class4 | -3242.72 | 8 | 6501.44 | 6547.03 |
| WAVE 8 (n=2,081) |  |  |  |  |
| class1 | -3631.24 | 2 | 7266.49 | 7277.77 |
| class2 | -3542.40 | 4 | 7092.80 | 7115.37 |
| class3 | -3267.15 | 6 | 6546.30 | 6580.15 |
| class4 | -3043.47 | 8 | 6102.94 | 6148.07 |

Notes: AIC=Akaike information criterion, BIC=Bayesian information criterion. df= degrees of freedom. n= number of observations. Apart from Wave 1, convergence (model identification) in the estimation of the number of classes were not achieved beyond four classes. This explains why a maximum of four classes were reported for other survey waves 2 to 8.

# Table S3: Definition of dependent and independent variables included in the multinomial logistic regression model

| Variable name | Definition and measurement |
| --- | --- |
| Dependent variable |  |
| Support for the NHS COVID-19 contact tracing app | Wave 1 to 4, four classes:  1=Not supportive  2=Ambivalent  3=Somewhat supportive  4=Completely supportive  Wave 5, three classes:  1= Not supportive  2=Ambivalent  3= Supportive  Wave 6 to 8, four classes:  1=Least supportive  2=Less supportive  3=Ambivalent  4=Supportive |
| Independent variables |  |
| Age | Age of respondents in years.  Measured as a numeric continuous variables |
| Gender | Gender of respondents  Dummy variable:  1 if the respondent is a female, 0 otherwise |
| Ethnicity | Ethnic background of respondents  Dummy variable:  1 if the respondent self-described as white, 0 otherwise |
| Self-reported health | General health as reported by the respondents  Dummy variable:  1 if the respondent described the reported their health a good/very good, 0 otherwise |
| Disability or health problem lasting (or expected to last) at least 12 months | Whether the respondent has a health problem or disability which limits their day-to-day activities  Dummy variable:  1 if the respondent reported day-to-day activities limited a lot or a little, 0 otherwise |
| Consider themselves vulnerable to COVID-19 | Whether respondent considers themselves to be vulnerable to COVID-19 because of their age, pre-existing health condition, pregnancy or another reason  Dummy variable:  1 if the respondent considers self be vulnerable to COVID-19, 0 otherwise |
| Region live | Region lives in England or Wales  Dummy variable:  1 if the respondent is located in the North (including Yorkshire), 0 otherwise;  1 if the respondent is located in Midlands and East of England, 0 otherwise;  1 if the respondent is located in Wales, 0 otherwise; and  1 if the respondent is located in London and the South, 0 otherwise |
| Household Income category | Household income bracket of respondents  Dummy variable:  1 if the respondent’s household income is Under £14,999, 0 otherwise;  1 if the respondent’s household income is between £15,000 - £24,999, 0 otherwise;  1 if the respondent’s household income is between £25,000 - £34,999, 0 otherwise;  1 if the respondent’s household income is between £35,000-£60,000, 0 otherwise; and  1 if the respondent’s household income is Over £60,000, 0 otherwise |
| House ownership | House tenure of respondent  Dummy variable:  1 if the respondent lives in own house, 0 otherwise;  1 if the respondent lives in rented house, a0 otherwise; and  1 if the respondent has another arrangement including living rent free with friend/family, 0 otherwise; |
| App installed at current survey wave | Whether NHS COVID-19 app is installed on their phone at current survey wave  Dummy variable:  1 if the respondent has app currently installed, 0 otherwise;  1 if the respondent has app currently uninstalled, 0 otherwise; and  1 if the respondent has never installed app , 0 otherwise; |
| COVID-19 infection status | Whether respondent has had COVID-19 infection since the previous survey  Dummy variable:  1 if the respondent reported definitely having had COVID-19 infection, 0 otherwise;  1 if the respondent reported have not had COVID-19 infection, 0 otherwise; and  1 if the respondent reported that they probably have had the infection or don’t know it had COVID-19 infection, 0 otherwise |
| Extent trust in government to control the spread of COVID-19 | The extent to which trust government to control the spread of COVID-19 infections  Dummy variable:  1 if the respondent reported having a great deal or a fair amount of trust, 0 otherwise; and  1 if the respondent reported having not very much trust, no trust or don’t know, 0 otherwise. |
| Risk of COVID-19 to self | Extent to which respondents is concerned about the risk COVID-19 poses to self  Dummy variable:  1 if the respondent is fairly concerned about the risk, 0 otherwise;  1 if the respondent is not very concerned about the risk, 0 otherwise;  1 if the respondent is not at all concerned about the risk, 0 otherwise; and  1 if the respondent is very concerned about the risk, 0 otherwise; |
| Risk of COVID-19 to country as a whole | Extent to which respondent is concerned about the risk COVID-19 poses to the country as a whole  Dummy variable:  1 if the respondent is very concerned about the risk, 0 otherwise;  1 if the respondent is fairly concerned about the risk, 0 otherwise;  1 if the respondent is not very concerned about the risk, 0 otherwise; and  1 if the respondent is not at all concerned about the risk, 0 otherwise |

**Table S4: Correlation matrix of independent variables (Wave 1 model)**

| **Variable** | Age | Gender (Female) | Ethnicity (White) | Self-reported health (good/very good)h | Disability (Limits a lot/a little) | Consider self-vulnerable to COVID-19 (Yes) | North (incl. York) | Mids & East of Eng | Wales | HH income Under £14,999 | HH income £15,000 - £24,999 | HH income £25,000 - £34,999 | HH income £35,000-£60,000 |
| --- | --- | --- | --- | --- | --- | --- | --- | --- | --- | --- | --- | --- | --- |
| Age | 1 |  |  |  |  |  |  |  |  |  |  |  |  |
| Gender (Female) | -0.1691 | 1 |  |  |  |  |  |  |  |  |  |  |  |
| Ethnicity (White) | 0.1086 | 0.0286 | 1 |  |  |  |  |  |  |  |  |  |  |
| Self-reported health (good/very good)h | -0.1336 | 0.0194 | -0.0179 | 1 |  |  |  |  |  |  |  |  |  |
| Disability (Limits a lot/a little) | 0.2000 | 0.0460 | 0.054 | -0.4859 | 1 |  |  |  |  |  |  |  |  |
| Consider self-vulnerable to COVID-19 (Yes) | 0.4817 | -0.0169 | 0.0293 | -0.3498 | 0.3702 | 1 |  |  |  |  |  |  |  |
| North (incl. York) | -0.0581 | -0.0513 | 0.1191 | -0.0297 | -0.0149 | 0.0109 | 1 |  |  |  |  |  |  |
| Mids & East of England | 0.0067 | -0.0072 | 0.0022 | -0.0139 | 0.0094 | 0.0043 | -0.3717 | 1 |  |  |  |  |  |
| Wales | 0.0319 | 0.0383 | -0.1365 | 0.0654 | -0.0234 | -0.0145 | -0.4895 | -0.4996 | 1 |  |  |  |  |
| HH income: Under £14,999 | 0.1106 | 0.0764 | 0.0261 | -0.1891 | 0.2309 | 0.1256 | 0.0254 | -0.0136 | -0.0122 | 1 |  |  |  |
| HH income: £15,000 - £24,999 | 0.0662 | -0.0003 | 0.0380 | -0.0598 | 0.0468 | 0.0604 | 0.0351 | 0.0111 | -0.0618 | -0.1446 | 1 |  |  |
| HH income: £25,000 - £34,999 | 0.0308 | -0.0300 | 0.0111 | -0.0078 | 0.0047 | -0.0012 | -0.0101 | 0.0345 | -0.0188 | -0.1340 | -0.154 | 1 |  |
| HH income: £35,000-£60,000 | -0.0785 | -0.0512 | 0.0348 | 0.0899 | -0.0691 | -0.0800 | 0.0177 | -0.0029 | -0.0012 | -0.1847 | -0.2124 | -0.197 | 1 |
| HH ownership: Own | 0.4124 | -0.0742 | 0.0772 | 0.0813 | -0.0632 | 0.1260 | -0.0269 | 0.0369 | -0.0205 | -0.1601 | -0.064 | -0.01 | 0.0761 |
| HH ownership: Rent | -0.1815 | 0.0222 | -0.0143 | -0.0800 | 0.1121 | -0.0195 | 0.0368 | -0.0526 | 0.0328 | 0.2097 | 0.0849 | 0.0159 | -0.0404 |
| App currently installed | -0.0004 | -0.0425 | 0.0248 | 0.0076 | -0.0207 | 0.0364 | -0.0297 | 0.0187 | 0.0422 | -0.0743 | -0.0223 | -0.021 | 0.0528 |
| App currently uninstalled | -0.0581 | 0.0491 | -0.0186 | 0.0049 | -0.0357 | -0.0636 | 0.0151 | 0.0006 | -0.0173 | -0.0047 | 0.0130 | -0.006 | -0.0123 |
| COVID-19 status: Definitely had | -0.0343 | 0.0137 | -0.0376 | 0.0276 | 0.0002 | -0.0086 | 0.0463 | -0.0272 | -0.0008 | -0.0337 | -0.0192 | -0.013 | 0.0671 |
| COVID-19 status: Haven't had | 0.1118 | 0.0416 | 0.0532 | 0.0064 | 0.0510 | 0.0617 | -0.0400 | 0.0165 | 0.0231 | 0.0104 | 0.0249 | -0.019 | -0.0085 |
| Extent trust govt (Great/Fair) | 0.1693 | -0.0271 | 0.0086 | -0.0057 | 0.0373 | 0.0669 | -0.0738 | -0.0260 | -0.0245 | 0.0479 | 0.0477 | -0.011 | -0.0253 |
| Extent concerned risk of COVID-19 to self: fairly | 0.0442 | 0.0724 | 0.024 | 0.0057 | -0.0496 | 0.0562 | -0.0455 | 0.0201 | 0.0382 | -0.0523 | -0.015 | 0.0088 | 0.029 |
| Extent concerned risk of COVID-19 to self: Not very | -0.1483 | -0.0537 | 0.0335 | 0.1321 | -0.1342 | -0.2294 | 0.0078 | -0.0082 | 0.0019 | -0.0101 | -0.0616 | -0.026 | 0.0497 |
| Extent concerned risk of COVID-19 to self: Not at all | -0.0929 | -0.0928 | 0.0307 | 0.0857 | -0.0627 | -0.1827 | 0.0245 | -0.0026 | -0.0205 | -0.0067 | 0.0471 | 0.0119 | -0.0177 |
| Extent concerned risk of COVID-19 to country: Fairly | -0.0757 | -0.0225 | 0.0065 | 0.0066 | -0.0778 | -0.1070 | -0.0036 | 0.0046 | 0.0074 | -0.0045 | -0.0178 | 0.0284 | 0.0234 |
| Extent concerned risk of COVID-19 to country: Not very | -0.0255 | -0.0976 | 0.0504 | 0.0603 | -0.0367 | -0.1092 | 0.0573 | -0.0242 | -0.0448 | -0.0203 | 0.0378 | -0.03 | 0.0243 |
| Extent concerned risk of COVID-19 to country: Not at all | -0.0136 | -0.0894 | 0.0146 | 0.0454 | -0.0236 | -0.0899 | -0.0117 | 0.0336 | -0.0178 | -0.0014 | 0.0278 | 0.0105 | -0.0205 |

Correlation matrix of independent variables (Wave 1 model) (continued)

| **Variable** | HH ownership: Own | HH ownership: Rent | App currently installed | App currently uninstalled | Definitely had COVID-19 | Haven't had COVID-19 | Great/Fair trust | Fairly concerned of risk to self | Not very concerned about risk to self | Not at all concerned about risk to self | Fairly concerned about risk to country | Not very concerned about risk to country | Not at all concerned about risk to country |
| --- | --- | --- | --- | --- | --- | --- | --- | --- | --- | --- | --- | --- | --- |
| HH ownership: Own | 1 |  |  |  |  |  |  |  |  |  |  |  |  |
| HH ownership: Rent | -0.7555 | 1 |  |  |  |  |  |  |  |  |  |  |  |
| App currently installed | 0.0011 | -0.0197 | 1 |  |  |  |  |  |  |  |  |  |  |
| App currently uninstalled | 0.0101 | -0.0131 | -0.4428 | 1 |  |  |  |  |  |  |  |  |  |
| COVID-19 status: Definitely had | -0.0503 | 0.0303 | -0.0474 | 0.0659 | 1 |  |  |  |  |  |  |  |  |
| COVID-19 status: Haven't had | 0.0995 | -0.0819 | 0.0356 | -0.0336 | -0.1661 | 1 |  |  |  |  |  |  |  |
| Extent trust govt (Great/Fair) | 0.0628 | -0.0116 | 0.0091 | 0.0094 | -0.0004 | 0.014 | 1 |  |  |  |  |  |  |
| Extent concerned risk of COVID-19 to self: fairly | 0.0484 | -0.0393 | 0.0847 | -0.0327 | -0.0326 | 0.0291 | 0.0116 | 1 |  |  |  |  |  |
| Extent concerned risk of COVID-19 to self: Not very | -0.0447 | 0.0121 | -0.0245 | 0.0331 | 0.0123 | -0.0431 | -0.0209 | -0.5469 | 1 |  |  |  |  |
| Extent concerned risk of COVID-19 to self: Not at all | -0.0204 | -0.011 | -0.1472 | 0.0421 | -0.0061 | -0.0208 | -0.0246 | -0.2576 | -0.1667 | 1 |  |  |  |
| Extent concerned risk of COVID-19 to country: Fairly | -0.0367 | 0.0183 | -0.0162 | 0.0275 | -0.0028 | -0.0179 | 0.0616 | 0.088 | 0.2506 | -0.0921 | 1 |  |  |
| Extent concerned risk of COVID-19 to country: Not very | 0.0305 | -0.0276 | -0.0957 | -0.0024 | 0.0114 | -0.0832 | -0.0147 | -0.2257 | 0.1976 | 0.3007 | -0.2239 | 1 |  |
| Extent concerned risk of COVID-19 to country: Not at all | 0.0178 | -0.0263 | -0.1025 | -0.0039 | 0.002 | -0.0447 | -0.0384 | -0.1493 | -0.0726 | 0.5444 | -0.1394 | -0.046 | 1 |

**Table S5: Correlation matrix of independent variables (Wave 5 model)**

| **Variable** | Age | Gender (Female) | Ethnicity  (White) | Self-reported health (good/very good)h | Disability (Limits a lot/a little) | Consider self-vulnerable to COVID-19 (Yes) | North (incl. York) | Mids & East of England | Wales | HH income Under £14,999 | HH income £15,000 - £24,999 | HH income £25,000 - £34,999 | HH income £35,000-£60,000 |
| --- | --- | --- | --- | --- | --- | --- | --- | --- | --- | --- | --- | --- | --- |
| Age | 1 |  |  |  |  |  |  |  |  |  |  |  |  |
| Gender (Female) | -0.1913 | 1 |  |  |  |  |  |  |  |  |  |  |  |
| Ethnicity (White) | 0.0978 | 0.0472 | 1 |  |  |  |  |  |  |  |  |  |  |
| Self-reported health (good/very good)h | -0.0915 | 0.0019 | 0.0098 | 1 |  |  |  |  |  |  |  |  |  |
| Disability (Limits a lot/a little) | 0.1852 | 0.0613 | 0.0571 | -0.4958 | 1 |  |  |  |  |  |  |  |  |
| Consider self-vulnerable to COVID-19 (Yes) | 0.4670 | -0.0210 | 0.0203 | -0.2854 | 0.363 | 1 |  |  |  |  |  |  |  |
| North (incl. York) | -0.0519 | -0.0543 | 0.1047 | -0.0089 | -0.0102 | 0.0225 | 1 |  |  |  |  |  |  |
| Mids & East of England | 0.0126 | 0.0110 | -0.0048 | -0.0451 | 0.0217 | 0.0066 | -0.3714 | 1 |  |  |  |  |  |
| Wales | 0.0321 | 0.0347 | -0.1214 | 0.0613 | -0.0406 | -0.0147 | -0.4865 | -0.4944 | 1 |  |  |  |  |
| HH income: Under £14,999 | 0.1074 | 0.0921 | 0.0284 | -0.2053 | 0.2258 | 0.1460 | 0.0226 | -0.0096 | -0.012 | 1 |  |  |  |
| HH income: £15,000 - £24,999 | 0.0781 | -0.0119 | 0.0428 | -0.0731 | 0.0550 | 0.0616 | 0.0257 | 0.0236 | -0.0641 | -0.1502 | 1 |  |  |
| HH income: £25,000 - £34,999 | 0.0151 | -0.0246 | 0.0288 | -0.0325 | -0.0022 | -0.0184 | -0.0126 | 0.0236 | -0.0066 | -0.1448 | -0.1639 | 1 |  |
| HH income: £35,000-£60,000 | -0.0992 | -0.0521 | 0.0114 | 0.0990 | -0.0693 | -0.0822 | -0.0018 | -0.0059 | 0.0176 | -0.1929 | -0.2183 | -0.2104 | 1 |
| HH ownership: Own | 0.3997 | -0.0994 | 0.0595 | 0.1042 | -0.0636 | 0.0901 | -0.0295 | 0.0411 | -0.0162 | -0.1720 | -0.0480 | -0.0016 | 0.0641 |
| HH ownership: Rent | -0.1800 | 0.0424 | -0.0083 | -0.1044 | 0.0998 | -0.0017 | 0.0516 | -0.0509 | 0.0205 | 0.2122 | 0.0753 | -0.0027 | -0.0386 |
| App currently installed | 0.0222 | -0.0414 | 0.0335 | 0.0594 | 0.0151 | 0.0489 | -0.0186 | -0.0032 | 0.0590 | -0.0638 | -0.0170 | -0.0116 | 0.0567 |
| App currently uninstalled | -0.0932 | 0.0649 | -0.0529 | -0.0063 | -0.0346 | -0.0820 | 0.0090 | 0.0128 | -0.0243 | -0.0013 | -0.0034 | -0.0239 | -0.0192 |
| COVID-19 status: Definitely had | -0.0375 | -0.0228 | -0.0147 | 0.0157 | -0.0131 | -0.0415 | 0.0110 | 0.0002 | 0.0144 | -0.0333 | 0.0060 | -0.0520 | 0.0160 |
| COVID-19 status: Haven't had | 0.0874 | 0.0615 | 0.0594 | 0.0164 | 0.0142 | 0.0551 | -0.0005 | 0.0027 | -0.0021 | -0.0046 | -0.0360 | 0.0416 | 0.0028 |
| Extent trust govt (Great/Fair) | 0.2321 | -0.0413 | 0.0113 | 0.0525 | 0.0261 | 0.0770 | -0.0527 | -0.0134 | 0.0096 | 0.0106 | 0.0870 | -0.0182 | -0.0278 |
| Extent concerned risk of COVID-19 to self: fairly | 0.0531 | 0.0542 | -0.0001 | -0.0368 | 0.0103 | 0.1059 | 0.0254 | -0.0136 | -0.0200 | -0.0022 | -0.0152 | 0.0078 | 0.0299 |
| Extent concerned risk of COVID-19 to self: Not very | -0.1082 | -0.0107 | 0.0778 | 0.1198 | -0.1362 | -0.2025 | -0.0183 | -0.0154 | 0.0446 | -0.0699 | -0.0316 | -0.0229 | 0.0065 |
| Extent concerned risk of COVID-19 to self: Not at all | -0.1000 | -0.0920 | -0.0252 | 0.0808 | -0.0755 | -0.1769 | -0.0198 | 0.0124 | 0.0138 | -0.0089 | 0.0139 | 0.0086 | 0.0030 |
| Extent concerned risk of COVID-19 to country: Fairly | -0.0922 | 0.0458 | 0.0201 | 0.0316 | -0.0880 | -0.0556 | 0.0178 | -0.0245 | 0.0170 | -0.0417 | -0.0586 | -0.0007 | 0.0238 |
| Extent concerned risk of COVID-19 to country: Not very | -0.0136 | -0.0931 | 0.0172 | 0.0698 | -0.0826 | -0.1213 | 0.0082 | -0.0112 | 0.0010 | -0.0083 | 0.0226 | -0.0067 | 0.0043 |
| Extent concerned risk of COVID-19 to country: Not at all | -0.0460 | -0.0680 | 0.0005 | 0.0369 | -0.0186 | -0.0928 | -0.0263 | 0.0315 | -0.0054 | -0.0032 | 0.0267 | 0.0096 | -0.0171 |

Correlation matrix of independent variables (Wave 5 model) (continued)

| **Variable** | HH ownership: Own | HH ownership: Rent | App currently installed | App currently uninstalled | COVID-19 status: Definitely had | COVID-19 status: Haven't had | Extent trust govt (Great/Fair) | Extent concerned risk of COVID-19 to self: fairly | Extent concerned risk of COVID-19 to self: Not very | Extent concerned risk of COVID-19 to self: Not at all | Extent concerned risk of COVID-19 to country: Fairly | Extent concerned risk of COVID-19 to country: Not very | Extent concerned risk of COVID-19 to country: Not at all |
| --- | --- | --- | --- | --- | --- | --- | --- | --- | --- | --- | --- | --- | --- |
| HH ownership: Own | 1 |  |  |  |  |  |  |  |  |  |  |  |  |
| HH ownership: Rent | -0.7669 | 1 |  |  |  |  |  |  |  |  |  |  |  |
| App currently installed | 0.0212 | -0.0232 | 1 |  |  |  |  |  |  |  |  |  |  |
| App currently uninstalled | -0.0096 | -0.0031 | -0.4600 | 1 |  |  |  |  |  |  |  |  |  |
| COVID-19 status: Definitely had | -0.0073 | -0.0003 | -0.0503 | 0.0198 | 1 |  |  |  |  |  |  |  |  |
| COVID-19 status: Haven't had | 0.0768 | -0.0695 | 0.0815 | -0.0289 | -0.3840 | 1 |  |  |  |  |  |  |  |
| Extent trust govt (Great/Fair) | 0.1051 | -0.0505 | 0.0249 | 0.0278 | 0.0003 | 0.0329 | 1 |  |  |  |  |  |  |
| Extent concerned risk of COVID-19 to self: fairly | 0.0109 | 0.0098 | 0.0902 | -0.0135 | -0.0342 | -0.0243 | 0.0071 | 1 |  |  |  |  |  |
| Extent concerned risk of COVID-19 to self: Not very | -0.0201 | -0.0112 | -0.0132 | 0.0237 | -0.0075 | 0.0145 | -0.0002 | -0.6052 | 1 |  |  |  |  |
| Extent concerned risk of COVID-19 to self: Not at all | -0.0094 | -0.0145 | -0.1444 | 0.0585 | 0.0751 | -0.0523 | -0.0769 | -0.2701 | -0.2034 | 1 |  |  |  |
| Extent concerned risk of COVID-19 to country: Fairly | -0.0423 | 0.0156 | -0.0406 | 0.0673 | -0.0184 | -0.0238 | 0.0691 | 0.1779 | 0.1902 | -0.1762 | 1 |  |  |
| Extent concerned risk of COVID-19 to country: Not very | 0.0211 | -0.0023 | -0.0531 | -0.0241 | 0.0271 | -0.0204 | 0.0081 | -0.2360 | 0.1985 | 0.2601 | -0.3633 | 1 |  |
| Extent concerned risk of COVID-19 to country: Not at all | 0.0287 | -0.0633 | -0.1008 | -0.0065 | 0.0114 | -0.0650 | -0.0712 | -0.1544 | -0.0750 | 0.5023 | -0.1941 | -0.0555 | 1 |

**Table S6: Correlation matrix of independent variables (Wave 8 model)**

| **Variables** | Age | Gender(Female) | Ethnicity (White) | Self-reported health (good/very good)h | Disability (Limits a lot/a little) | Consider self-vulnerable to COVID-19 (Yes) | North (incl. York) | Mids & East of England | Wales | HH income Under £14,999 | HH income £15,000 - £24,999 | HH income £25,000 - £34,999 | HH income £35,000-£60,000 |
| --- | --- | --- | --- | --- | --- | --- | --- | --- | --- | --- | --- | --- | --- |
| Age | 1 |  |  |  |  |  |  |  |  |  |  |  |  |
| Gender (Female) | -0.1460 | 1 |  |  |  |  |  |  |  |  |  |  |  |
| Ethnicity (White) | 0.0895 | 0.0764 | 1 |  |  |  |  |  |  |  |  |  |  |
| Self-reported health (good/very good)h | -0.0879 | -0.0189 | -0.0257 | 1 |  |  |  |  |  |  |  |  |  |
| Disability (Limits a lot/a little)h | 0.1611 | 0.0248 | 0.0547 | -0.5189 | 1 |  |  |  |  |  |  |  |  |
| Consider self-vulnerable to COVID-19 (Yes) | 0.4729 | -0.0050 | 0.0208 | -0.3230 | 0.3803 | 1 |  |  |  |  |  |  |  |
| North (incl. York) | -0.0713 | -0.0620 | 0.0819 | -0.0368 | -0.0173 | -0.0051 | 1 |  |  |  |  |  |  |
| Mids & East of England | 0.0050 | -0.0111 | 0.0054 | -0.0176 | 0.0333 | -0.0033 | -0.3748 | 1 |  |  |  |  |  |
| Wales | 0.0437 | 0.0671 | -0.1193 | 0.0683 | -0.0453 | 0.0056 | -0.4877 | -0.4867 | 1 |  |  |  |  |
| HH income: Under £14,999 | 0.1048 | 0.0837 | 0.0152 | -0.1901 | 0.1761 | 0.1385 | 0.0383 | -0.0062 | -0.0287 | 1 |  |  |  |
| HH income: £15,000 - £24,999 | 0.0467 | 0.0318 | 0.0215 | -0.0386 | 0.0716 | 0.0394 | 0.0345 | -0.0014 | -0.0488 | -0.1608 | 1 |  |  |
| HH income: £25,000 - £34,999 | -0.0078 | -0.0146 | 0.0344 | -0.0194 | 0.0269 | -0.0176 | -0.0176 | 0.0361 | -0.0127 | -0.1587 | -0.1763 | 1 |  |
| HH income: £35,000-£60,000 | -0.0679 | -0.0367 | -0.0223 | 0.0910 | -0.0612 | -0.0691 | -0.0203 | -0.0007 | 0.0118 | -0.1923 | -0.2137 | -0.2109 | 1 |
| HH ownership: Own | 0.3538 | -0.0668 | 0.0451 | 0.1571 | -0.0849 | 0.0569 | -0.0267 | 0.0231 | -0.0193 | -0.1825 | -0.0685 | -0.0318 | 0.0778 |
| HH ownership: Rent | -0.1460 | 0.0266 | -0.0169 | -0.1574 | 0.1110 | 0.0158 | 0.0530 | -0.0403 | 0.0266 | 0.2230 | 0.0746 | 0.0110 | -0.0621 |
| App currently installed | 0.0360 | -0.0554 | -0.0078 | 0.0054 | 0.0327 | 0.0676 | -0.0099 | 0.0183 | 0.0130 | -0.0705 | -0.0019 | -0.0171 | 0.0239 |
| App currently uninstalled | -0.1061 | 0.0725 | -0.0322 | 0.0724 | -0.0561 | -0.0868 | 0.0176 | -0.0050 | 0.00570 | -0.0203 | -0.0247 | -0.0202 | 0.0019 |
| COVID-19 status: Definitely had | -0.0479 | 0.0462 | 0.0371 | 0.0072 | -0.0441 | -0.0288 | 0.0319 | 0.0010 | -0.0309 | -0.0340 | -0.0591 | 0.0116 | 0.0273 |
| COVID-19 status: Haven't had | 0.1041 | 0.0058 | -0.0075 | 0.0156 | 0.0285 | 0.0607 | -0.0075 | 0.0108 | 0.0143 | 0.0094 | 0.0186 | 0.0262 | 0.0042 |
| Extent trust govt (Great/Fair) | 0.1845 | -0.0647 | -0.0044 | 0.0290 | 0.0440 | 0.0536 | -0.0588 | -0.0260 | -0.0045 | -0.0013 | 0.0938 | -0.0316 | 0.0449 |
| Extent concerned risk of COVID-19 to self: fairly | 0.0673 | 0.1276 | 0.0130 | -0.0126 | -0.0375 | 0.0767 | -0.0209 | 0.0111 | 0.0036 | -0.0040 | -0.0022 | -0.0220 | 0.0221 |
| Extent concerned risk of COVID-19 to self: Not very | -0.0994 | -0.0753 | 0.0165 | 0.1390 | -0.1686 | -0.2269 | -0.0080 | -0.0069 | 0.0532 | -0.0287 | -0.0442 | 0.0033 | 0.0289 |
| Extent concerned risk of COVID-19 to self: Not at all | -0.0704 | -0.0993 | 0.0319 | 0.0924 | -0.0520 | -0.1584 | -0.0160 | 0.0313 | -0.0325 | -0.0014 | 0.0019 | 0.0299 | -0.0012 |
| Extent concerned risk of COVID-19 to country: Fairly | 0.0076 | 0.0807 | 0.0295 | 0.0180 | -0.0746 | -0.0328 | -0.0240 | -0.0145 | 0.0578 | 0.0035 | -0.0199 | -0.0148 | 0.0240 |
| Extent concerned risk of COVID-19 to country: Not very | -0.0955 | -0.1065 | 0.0035 | 0.1104 | -0.1237 | -0.1988 | 0.0127 | 0.0029 | -0.0146 | -0.0531 | -0.0196 | 0.0041 | -0.0013 |
| Extent concerned risk of COVID-19 to country: Not at all | -0.0465 | -0.0851 | 0.0427 | 0.0415 | -0.0045 | -0.0847 | -0.0110 | 0.0539 | -0.0435 | 0.0052 | 0.0242 | 0.0034 | -0.0161 |

Correlation matrix of independent variables (Wave 8 model) (continued)

| **Variable** | Own house | Rent | App currently installed | App currently uninstalled | COVID-19 status: Definitely had | COVID-19 status: Haven't had | Extent trust govt (Great/Fair) | Extent concerned risk of COVID-19 to self: fairly | Extent concerned risk of COVID-19 to self: Not very | Extent concerned risk of COVID-19 to self: Not at all | Extent concerned risk of COVID-19 to country: Fairly | Extent concerned risk of COVID-19 to country: Not very | Extent concerned risk of COVID-19 to country: Not at all |
| --- | --- | --- | --- | --- | --- | --- | --- | --- | --- | --- | --- | --- | --- |
| HH ownership: Own | 1 |  |  |  |  |  |  |  |  |  |  |  |  |
| HH ownership: Rent | -0.8010 | 1 |  |  |  |  |  |  |  |  |  |  |  |
| App currently installed | 0.0486 | -0.0477 | 1 |  |  |  |  |  |  |  |  |  |  |
| App currently uninstalled | -0.002 | -0.0050 | -0.4523 | 1 |  |  |  |  |  |  |  |  |  |
| COVID-19 status: Definitely had | -0.009 | -0.0076 | -0.0129 | 0.0383 | 1 |  |  |  |  |  |  |  |  |
| COVID-19 status: Haven't had | 0.0649 | -0.0517 | 0.0740 | -0.0480 | -0.5296 | 1 |  |  |  |  |  |  |  |
| Extent trust govt (Great/Fair) | 0.0909 | -0.0452 | 0.0129 | 0.0207 | 0.0239 | 0.0284 | 1 |  |  |  |  |  |  |
| Extent concerned risk of COVID-19 to self: fairly | 0.0361 | -0.0431 | 0.1397 | -0.058 | -0.0580 | 0.0323 | 0.0218 | 1 |  |  |  |  |  |
| Extent concerned risk of COVID-19 to self: Not very | -0.001 | 0.0023 | -0.0742 | 0.0856 | 0.0829 | -0.0298 | 0.0062 | -0.569 | 1 |  |  |  |  |
| Extent concerned risk of COVID-19 to self: Not at all | -0.002 | 0.0135 | -0.1479 | 0.0014 | 0.0327 | -0.1018 | -0.023 | -0.283 | -0.1921 | 1 |  |  |  |
| Extent concerned risk of COVID-19 to country: Fairly | 0.0015 | -0.0033 | 0.0689 | 0.0079 | -0.0198 | 0.0329 | 0.0741 | 0.3697 | 0.0296 | -0.2399 | 1 |  |  |
| Extent concerned risk of COVID-19 to country: Not very | -0.015 | 0.0203 | -0.1184 | 0.0668 | 0.0895 | -0.0809 | 0.0110 | -0.345 | 0.4371 | 0.1793 | -0.446 | 1 |  |
| Extent concerned risk of COVID-19 to country: Not at all | 0.0118 | -0.0140 | -0.1187 | -0.014 | -0.0031 | -0.0965 | -0.054 | -0.195 | -0.1053 | 0.6472 | -0.227 | -0.0893 | 1 |

# Table S*7:* Variance inflation factor of predictors (Wave 1 model)

| **Variable** | **VIF** | **1/VIF** |
| --- | --- | --- |
| Age | 1.81 | 0.5528 |
| Gender(Female) | 1.1 | 0.9057 |
| Ethnicity (White) | 1.08 | 0.9278 |
| Self-reported health (good/very good)h | 1.43 | 0.6989 |
| Disability (Limits a lot/a little) | 1.48 | 0.6758 |
| Consider self-vulnerable to COVID-19 (Yes) | 1.64 | 0.6113 |
| North (including Yorkshire) | 4.24 | 0.2361 |
| Midlands and East of England | 4.26 | 0.2348 |
| Wales | 4.9 | 0.2040 |
| HH income: Under £14,999 | 1.32 | 0.7563 |
| HH income: £15,000 - £24,999 | 1.23 | 0.8145 |
| HH income: £25,000 - £34,999 | 1.17 | 0.8555 |
| HH income: £35,000-£60,000 | 1.23 | 0.8140 |
| HH ownership: Own | 2.96 | 0.3380 |
| HH ownership: Rent | 2.54 | 0.3940 |
| App currently installed | 1.32 | 0.7584 |
| App currently uninstalled | 1.27 | 0.7864 |
| COVID-19 status: Definitely had | 1.05 | 0.9534 |
| COVID-19 status: Haven't had | 1.07 | 0.9352 |
| Extent trust govt (Great/Fair) | 1.11 | 0.9039 |
| Extent concerned risk of COVID-19 to self: fairly | 2.09 | 0.4791 |
| Extent concerned risk of COVID-19 to self: Not very | 2.52 | 0.3972 |
| Extent concerned risk of COVID-19 to self: Not at all | 2.38 | 0.4207 |
| Extent concerned risk of COVID-19 to country: Fairly | 1.38 | 0.7265 |
| Extent concerned risk of COVID-19 to country: Not very | 1.52 | 0.6583 |
| Extent concerned risk of COVID-19 to country: Not at all | 1.66 | 0.6012 |
| Mean VIF | 1.91 |  |

# Table S8: Variance inflation factor of predictors (VIF) (Wave 5 model)

| **Variable** | **VIF** | **1/VIF** |
| --- | --- | --- |
| Age | 1.83 | 0.5475 |
| Gender (Female) | 1.11 | 0.8972 |
| Ethnicity (White) | 1.08 | 0.9288 |
| Self-reported health (good/very good) | 1.42 | 0.7019 |
| Has disability | 1.5 | 0.6647 |
| Vulnerable | 1.56 | 0.6401 |
| North (including Yorkshire) | 3.87 | 0.2586 |
| Midlands and East of England | 3.89 | 0.2574 |
| Wales | 4.46 | 0.2240 |
| HH income: Under £14,999 | 1.35 | 0.7386 |
| HH income: £15,000 - £24,999 | 1.26 | 0.7953 |
| HH income: £25,000 - £34,999 | 1.2 | 0.8316 |
| HH income: £35,000-£60,000 | 1.25 | 0.7978 |
| House ownership: Own | 3.03 | 0.3304 |
| House ownership: Rent | 2.63 | 0.3801 |
| App currently installed | 1.36 | 0.7335 |
| App currently uninstalled | 1.31 | 0.7614 |
| COVID-19 status: Definitely had | 1.19 | 0.8374 |
| COVID-19 status: Haven't had | 1.22 | 0.8223 |
| Extent trust govt (Great/Fair) | 1.11 | 0.8986 |
| Extent concerned risk of COVID-19 to self: fairly | 2.68 | 0.3728 |
| Extent concerned risk of COVID-19 to self: Not very | 3.13 | 0.3198 |
| Extent concerned risk of COVID-19 to self: Not at all | 2.38 | 0.4206 |
| Extent concerned risk of COVID-19 to country: Fairly | 1.66 | 0.6030 |
| Extent concerned risk of COVID-19 to country: Not very | 1.63 | 0.6130 |
| Extent concerned risk of COVID-19 to country: Not at all | 1.58 | 0.6334 |
| Mean VIF | 1.95 |  |

# Table S9: Variance inflation factor of predictors (VIF) (Wave 8 model)

| **Variable** | **VIF** | **1/VIF** |
| --- | --- | --- |
| Age | 1.75 | 0.5719 |
| Gender (Female) | 1.12 | 0.8957 |
| Ethnicity (White) | 1.06 | 0.9436 |
| Self-reported health (good/very good) | 1.48 | 0.6752 |
| Has disability | 1.56 | 0.6423 |
| Vulnerable | 1.64 | 0.6081 |
| North (including Yorkshire) | 3.9 | 0.2562 |
| Midlands and East of England | 3.87 | 0.2586 |
| Wales | 4.44 | 0.2250 |
| HH income: Under £14,999 | 1.36 | 0.7334 |
| HH income: £15,000 - £24,999 | 1.28 | 0.7839 |
| HH income: £25,000 - £34,999 | 1.23 | 0.8145 |
| HH income: £35,000-£60,000 | 1.26 | 0.7945 |
| HH ownership: Own | 3.44 | 0.2903 |
| HH ownership: Rent | 3.06 | 0.3272 |
| App currently installed | 1.35 | 0.7403 |
| App currently uninstalled | 1.31 | 0.7635 |
| COVID-19 status: Definitely had | 1.44 | 0.6965 |
| COVID-19 status: Haven't had | 1.45 | 0.6897 |
| Extent trust govt (Great/Fair) | 1.11 | 0.9044 |
| Extent concerned risk of COVID-19 to self: fairly | 2.82 | 0.3551 |
| Extent concerned risk of COVID-19 to self: Not very | 3.54 | 0.2827 |
| Extent concerned risk of COVID-19 to self: Not at all | 3.03 | 0.3295 |
| Extent concerned risk of COVID-19 to country: Fairly | 2.07 | 0.4838 |
| Extent concerned risk of COVID-19 to country: Not very | 2.44 | 0.4095 |
| Extent concerned risk of COVID-19 to country: Not at all | 2.25 | 0.4448 |
| Mean VIF | 2.12 |  |

**Table S10: Characteristics of individual belonging to identified subgroups of support for NHS COVID-19 app (survey waves 1 – 4 (14^th^ October 2020 to 15^th^ February 2021))**

|  | **Not supportive**  **(24% (n=1765))** | | **Ambivalent**  **(29% (n=2124)** | | **Somewhat supportive (20% (n=1421)** | | **Completely supportive (26% (n=1900)** | | **Total**  **(n=7210)** | | **Chi^2^ value** | **P value** |
| --- | --- | --- | --- | --- | --- | --- | --- | --- | --- | --- | --- | --- |
|  | **Number** | **%** | **Number** | **%** | **Number** | **%** | **Number** | **%** | **Number** | **%** |  |  |
| **Support for NHS COVID-19 app** | | | | | | | | | | | | |
| 1. Not supportive at all | 902 | 51% | 0 | 0% | 0 | 0% | 0 | 0% | 902 | 13% | 2.20E+04 | 0.000 |
| 2 | 863 | 49% | 0 | 0% | 0 | 0% | 0 | 0% | 863 | 12% |  |  |
| 3 | 0 | 0% | 2,124 | 100% | 0 | 0% | 0 | 0% | 2,124 | 29% |  |  |
| 4 | 0 | 0% | 0 | 0% | 1,421 | 100% | 0 | 0% | 1,421 | 20% |  |  |
| 5. Completely support | 0 | 0% | 0 | 0% | 0 | 0% | 1,900 | 100% | 1,900 | 26% |  |  |
| **General health** | | | | | | | | | | | | |
| Very good | 375 | 21% | 427 | 20% | 310 | 22% | 527 | 28% | 1,639 | 23% | 55.99 | 0.000 |
| Good | 913 | 52% | 1,134 | 53% | 754 | 53% | 871 | 46% | 3,672 | 51% |  |  |
| Fair | 334 | 19% | 408 | 19% | 261 | 18% | 343 | 18% | 1,346 | 19% |  |  |
| Bad | 84 | 5% | 103 | 5% | 65 | 5% | 116 | 6% | 368 | 5% |  |  |
| Very bad | 20 | 1% | 22 | 1% | 5 | 0% | 22 | 1% | 69 | 1% |  |  |
| Missing | 39 | 2% | 30 | 1% | 26 | 2% | 21 | 1% | 116 | 2% |  |  |
| **Disability** |  |  |  |  |  |  |  |  |  |  |  |  |
| Yes, limited a lot | 149 | 8% | 179 | 8% | 93 | 7% | 164 | 9% | 585 | 8% | 34.10 | 0.000 |
| Yes, limited a little | 249 | 14% | 313 | 15% | 236 | 17% | 354 | 19% | 1,152 | 16% |  |  |
| No | 1,317 | 75% | 1,598 | 75% | 1,060 | 75% | 1,359 | 72% | 5,334 | 74% |  |  |
| Prefer not to say | 39 | 2% | 23 | 1% | 15 | 1% | 22 | 1% | 99 | 1% |  |  |
| Missing | 11 | 1% | 11 | 1% | 17 | 1% | 1 | 0% | 40 | 1% |  |  |
| **Vulnerability to COVID-19 infection** | | | | | | | | | | | | |
| No | 1,039 | 59% | 1,258 | 59% | 815 | 57% | 920 | 48% | 4,032 | 56% | 79.60 | 0.000 |
| Yes | 682 | 39% | 834 | 39% | 584 | 41% | 960 | 51% | 3,060 | 42% |  |  |
| Missing | 44 | 2% | 32 | 2% | 22 | 2% | 20 | 1% | 118 | 2% |  |  |
| **Region** |  |  |  |  |  |  |  |  |  |  |  |  |
| North (incl. Yorkshire | 510 | 26% | 583 | 30% | 351 | 18% | 498 | 26% | 1,942 | 100% | 23.27 | 0.006 |
| Midlands & East of En | 502 | 25% | 558 | 28% | 415 | 21% | 517 | 26% | 1,992 | 100% |  |  |
| London & South | 635 | 22% | 842 | 30% | 589 | 21% | 758 | 27% | 2,824 | 100% |  |  |
| Wales | 118 | 26% | 141 | 31% | 66 | 15% | 127 | 28% | 452 | 100% |  |  |
| **Household income** | | | | | | | | | | | | |
| Under £14,999 | 231 | 13% | 232 | 11% | 154 | 11% | 208 | 11% | 825 | 11% | 64.26 | 0.000 |
| £15,000 - £24,999 | 217 | 12% | 297 | 14% | 187 | 13% | 333 | 18% | 1,034 | 14% |  |  |
| £25,000 - £34,999 | 232 | 13% | 274 | 13% | 184 | 13% | 226 | 12% | 916 | 13% |  |  |
| £35,000-£60,000 | 383 | 22% | 475 | 22% | 301 | 21% | 384 | 20% | 1,543 | 21% |  |  |
| Over £60,000 | 269 | 15% | 300 | 14% | 281 | 20% | 353 | 19% | 1,203 | 17% |  |  |
| Prefer not to say/Did | 433 | 25% | 546 | 26% | 314 | 22% | 396 | 21% | 1,689 | 23% |  |  |
| **Tenure** |  |  |  |  |  |  |  |  |  |  |  |  |
| Own | 1,015 | 58% | 1,181 | 56% | 831 | 58% | 1,213 | 64% | 4,240 | 59% | 64.28 | 0.000 |
| Rent | 506 | 29% | 634 | 30% | 418 | 29% | 497 | 26% | 2,055 | 29% |  |  |
| Live with friends or family | 194 | 11% | 289 | 14% | 146 | 10% | 168 | 9% | 797 | 11% |  |  |
| Other | 50 | 3% | 20 | 1% | 26 | 2% | 22 | 1% | 118 | 2% |  |  |
| **App installation** | | | | | | | | | | | | |
| currently installed | 367 | 21% | 789 | 37% | 845 | 59% | 1,409 | 74% | 3,410 | 47% | 1.50 | 0.000 |
| currently uninstalled | 305 | 17% | 400 | 19% | 299 | 21% | 313 | 16% | 1,317 | 18% |  |  |
| never installed | 1,093 | 62% | 935 | 44% | 277 | 19% | 178 | 9% | 2,483 | 34% |  |  |
| **Trust in government** | | | | | | | | | | | | |
| A great deal | 52 | 3% | 66 | 3% | 60 | 4% | 247 | 13% | 425 | 6% | 650.58 | 0.000 |
| A fair amount | 277 | 16% | 568 | 27% | 451 | 32% | 646 | 34% | 1,942 | 27% |  |  |
| Not very much | 517 | 29% | 803 | 38% | 521 | 37% | 563 | 30% | 2,404 | 33% |  |  |
| Not at all | 854 | 48% | 612 | 29% | 359 | 25% | 410 | 22% | 2,235 | 31% |  |  |
| Don’t know | 65 | 4% | 75 | 4% | 30 | 2% | 34 | 2% | 204 | 3% |  |  |
| Total | 1,765 | 100% | 2,124 | 100% | 1,421 | 100% | 1,900 | 100% | 7,210 | 100% |  |  |
| **Ethnicity** |  |  |  |  |  |  |  |  |  |  |  |  |
| White | 1,557 | 25% | 1,826 | 29% | 1,258 | 20% | 1,714 | 27% | 6,355 | 100% | 17.57 | 0.001 |
| Other | 208 | 24% | 298 | 35% | 163 | 19% | 186 | 22% | 855 | 100% |  |  |
| **Sex** |  |  |  |  |  |  |  |  |  |  |  |  |
| Male | 859 | 27% | 843 | 26% | 640 | 20% | 890 | 28% | 3,232 | 100% | 36.34 | 0.000 |
| Female | 906 | 23% | 1,281 | 33% | 781 | 20% | 1,010 | 25% | 3,978 | 100% |  |  |

**Table S11: Characteristics of individuals belonging to identified subgroups of support for NHS COVID-19 app (survey wave 5 (15- 31 March 2021))**

|  | **Subgroup 1:**  **Not supportive (34% (n=549)** | | **Subgroup 2:**  **Ambivalent (31% (n=497))** | | **Subgroup 3:**  **Supportive (35% (n=567)** | | **Total**  **(n=1613)** | | **Ch^2^ value** | **P-value** |
| --- | --- | --- | --- | --- | --- | --- | --- | --- | --- | --- |
|  | **Number** | **%** | **Number** | **%** | **Number** | **%** | **Number** | **%** |  |  |
| **App support** |  |  |  |  |  |  |  |  |  |  |
| 1-Not supportive at | 288 | 52% | 0 | 0% | 0 | 0% | 288 | 18% | 3.20E+03 | 0.000 |
| 2 | 261 | 48% | 0 | 0% | 0 | 0% | 261 | 16% |  |  |
| 3 | 0 | 0% | 497 | 100% | 0 | 0% | 497 | 31% |  |  |
| 4 | 0 | 0% | 0 | 0% | 258 | 46% | 258 | 16% |  |  |
| 5 -Completely support | 0 | 0% | 0 | 0% | 309 | 54% | 309 | 19% |  |  |
| **General health** |  |  |  |  |  |  |  |  |  |  |
| Very good | 124 | 23% | 82 | 16% | 128 | 23% | 334 | 21% | 13.12 | 0.108 |
| Good | 268 | 49% | 275 | 55% | 278 | 49% | 821 | 51% |  |  |
| Fair | 107 | 19% | 102 | 21% | 108 | 19% | 317 | 20% |  |  |
| Bad | 34 | 6% | 24 | 5% | 40 | 7% | 98 | 6% |  |  |
| Very bad | 4 | 1% | 7 | 1% | 4 | 1% | 15 | 1% |  |  |
| Missing | 12 | 2% | 7 | 1% | 9 | 2% | 28 | 2% |  |  |
| **Disability** |  |  |  |  |  |  |  |  |  |  |
| Yes, limited a lot | 47 | 9% | 36 | 7% | 50 | 9% | 133 | 8% | 4.02 | 0.674 |
| Yes, limited a little | 78 | 14% | 82 | 16% | 101 | 18% | 261 | 16% |  |  |
| No | 413 | 75% | 374 | 75% | 406 | 72% | 1,193 | 74% |  |  |
| Prefer not to say | 7 | 1% | 5 | 1% | 6 | 1% | 18 | 1% |  |  |
| Missing | 4 | 1% | 0 | 0% | 4 | 1% | 8 | 0% |  |  |
| **Vulnerability to COVID-19 infections** | | | | | | | | | | |
| No | 310 | 56% | 281 | 57% | 293 | 52% | 884 | 55% | 3.85 | 0.145 |
| Yes | 228 | 42% | 210 | 42% | 267 | 47% | 705 | 44% |  |  |
| Missing | 11 | 2% | 6 | 1% | 7 | 1% | 24 | 1% |  |  |
| **Region** |  |  |  |  |  |  |  |  |  |  |
| North (incl. Yorkshire | 154 | 36% | 130 | 30% | 148 | 34% | 432 | 100% | 7.94 | 0.242 |
| Midlands & East of En | 154 | 35% | 141 | 32% | 148 | 33% | 443 | 100% |  |  |
| London & South | 196 | 31% | 197 | 31% | 240 | 38% | 633 | 100% |  |  |
| Wales | 45 | 43% | 29 | 28% | 31 | 30% | 105 | 100% |  |  |
| **Household income** |  |  |  |  |  |  |  |  |  |  |
| Under £14,999 | 71 | 13% | 56 | 11% | 62 | 11% | 189 | 12% | 8.21 | 0.608 |
| £15,000 - £24,999 | 70 | 13% | 75 | 15% | 89 | 16% | 234 | 15% |  |  |
| £25,000 - £34,999 | 75 | 14% | 62 | 12% | 78 | 14% | 215 | 13% |  |  |
| £35,000-£60,000 | 121 | 22% | 103 | 21% | 127 | 22% | 351 | 22% |  |  |
| Over £60,000 | 92 | 17% | 78 | 16% | 102 | 18% | 272 | 17% |  |  |
| Prefer not to say/Did | 120 | 22% | 123 | 25% | 109 | 19% | 352 | 22% |  |  |
| **Tenure** |  |  |  |  |  |  |  |  |  |  |
| own | 327 | 60% | 289 | 58% | 348 | 61% | 964 | 60% | 5.21 | 0.517 |
| rent | 149 | 27% | 151 | 30% | 152 | 27% | 452 | 28% |  |  |
| Live with friends or family | 59 | 11% | 51 | 10% | 59 | 10% | 169 | 10% |  |  |
| other | 14 | 3% | 6 | 1% | 8 | 1% | 28 | 2% |  |  |
| **App installation** |  |  |  |  |  |  |  |  |  |  |
| Currently installed | 132 | 24% | 205 | 41% | 413 | 73% | 750 | 46% | 328.06 | 0.000 |
| Currently uninstalled | 109 | 20% | 106 | 21% | 99 | 17% | 314 | 19% |  |  |
| Never installed | 308 | 56% | 186 | 37% | 55 | 10% | 549 | 34% |  |  |
| **Trust in government** |  |  |  |  |  |  |  |  |  |  |
| A great deal | 24 | 4% | 32 | 6% | 82 | 14% | 138 | 9% | 143.32 | 0.000 |
| A fair amount | 149 | 27% | 202 | 41% | 236 | 42% | 587 | 36% |  |  |
| Not very much | 168 | 31% | 166 | 33% | 168 | 30% | 502 | 31% |  |  |
| Not at all | 193 | 35% | 76 | 15% | 69 | 12% | 338 | 21% |  |  |
| Don’t know | 15 | 3% | 21 | 4% | 12 | 2% | 48 | 3% |  |  |
| **Ethnicity** |  |  |  |  |  |  |  |  |  |  |
| White | 480 | 34% | 432 | 30% | 516 | 36% | 1,428 | 100% | 5.34 | 0.069 |
| Other | 69 | 37% | 65 | 35% | 51 | 28% | 185 | 100% |  |  |
| **Sex** |  |  |  |  |  |  |  |  |  |  |
| Male | 268 | 37% | 191 | 26% | 262 | 36% | 721 | 100% | 12.18 | 0.002 |
| Female | 281 | 32% | 306 | 34% | 305 | 34% | 892 | 100% |  |  |

**Table S12: Characteristics of individuals belonging to identified subgroups of support for NHS COVID-19 app (survey wave 6-8 (1^st^ July 2021 to 13^th^ December 2021))**

|  | **Subgroup 1:**  **Least supportive (23% (n=1568))** | | **Subgroup 2:**  **Less supportive (17% (n=1179))** | | **Subgroup 3:**  **Ambivalent (30% (n=2105))** | | **Subgroup 4: Supportive (30% (n=2100))** | | **Total**  **(n=6952)** | | **Chi-2** | **P-value** |
| --- | --- | --- | --- | --- | --- | --- | --- | --- | --- | --- | --- | --- |
|  | **Number** | **%** | **Number** | **%** | **Number** | **%** | **Number** | **%** | **Number** | **%** |  |  |
| App support |  |  |  |  |  |  |  |  |  |  |  |  |
| 1-Not supportive at | 1,568 | 100% | 0 | 0% | 0 | 0% | 0 | 0% | 1,568 | 23% | 2.10E+04 | 0.000 |
| 2 | 0 | 0% | 1,179 | 100% | 0 | 0% | 0 | 0% | 1,179 | 17% |  |  |
| 3 | 0 | 0% | 0 | 0% | 2,105 | 100% | 0 | 0% | 2,105 | 30% |  |  |
| 4 | 0 | 0% | 0 | 0% | 0 | 0% | 999 | 48% | 999 | 14% |  |  |
| 5 -Completely support | 0 | 0% | 0 | 0% | 0 | 0% | 1,101 | 52% | 1,101 | 16% |  |  |
| General health |  |  |  |  |  |  |  |  |  |  |  |  |
| Very good | 367 | 23% | 257 | 22% | 387 | 18% | 404 | 19% | 1,415 | 20% |  |  |
| Good | 745 | 48% | 607 | 51% | 1,043 | 50% | 1,016 | 48% | 3,411 | 49% |  |  |
| Fair | 301 | 19% | 223 | 19% | 492 | 23% | 452 | 22% | 1,468 | 21% | 52.8714 | 0.000 |
| Bad | 99 | 6% | 69 | 6% | 106 | 5% | 171 | 8% | 445 | 6% |  |  |
| Very bad | 28 | 2% | 8 | 1% | 20 | 1% | 33 | 2% | 89 | 1% |  |  |
| Missing | 28 | 2% | 15 | 1% | 57 | 3% | 24 | 1% | 124 | 2% |  |  |
| Disability |  |  |  |  |  |  |  |  |  |  |  |  |
| Yes, limited a lot | 155 | 10% | 79 | 7% | 194 | 9% | 257 | 12% | 685 | 10% |  |  |
| Yes, limited a little | 257 | 16% | 181 | 15% | 373 | 18% | 373 | 18% | 1,184 | 17% | 49.2267 | 0.000 |
| No | 1,109 | 71% | 903 | 77% | 1,484 | 70% | 1,444 | 69% | 4,940 | 71% |  |  |
| Prefer not to say | 36 | 2% | 11 | 1% | 36 | 2% | 20 | 1% | 103 | 1% |  |  |
| Missing | 11 | 1% | 5 | 0% | 18 | 1% | 6 | 0% | 40 | 1% |  |  |
| Vulnerability to COVID-19 |  |  |  |  |  |  |  |  |  |  |  |  |
| No | 475 | 30% | 435 | 37% | 701 | 33% | 542 | 26% | 2,153 | 31% |  |  |
| Yes | 368 | 23% | 252 | 21% | 530 | 25% | 662 | 32% | 1,812 | 26% | 79.3953 | 0.000 |
| Missing | 725 | 46% | 492 | 42% | 874 | 42% | 896 | 43% | 2,987 | 43% |  |  |
| Region |  |  |  |  |  |  |  |  |  |  |  |  |
| North (incl. Yorkshire | 384 | 23% | 288 | 18% | 473 | 29% | 494 | 30% | 1,639 | 100% |  |  |
| Midlands & East of En | 383 | 23% | 283 | 17% | 535 | 32% | 464 | 28% | 1,665 | 100% |  |  |
| London & South | 483 | 21% | 417 | 18% | 714 | 30% | 733 | 31% | 2,347 | 100% | 19.8535 | 0.019 |
| Wales | 318 | 24% | 191 | 15% | 383 | 29% | 409 | 31% | 1,301 | 100% |  |  |
| Household income |  |  |  |  |  |  |  |  |  |  |  |  |
| Under £14,999 | 187 | 12% | 128 | 11% | 273 | 13% | 253 | 12% | 841 | 12% |  |  |
| £15,000 - £24,999 | 225 | 14% | 173 | 15% | 364 | 17% | 342 | 16% | 1,104 | 16% | 36.8522 | 0.001 |
| £25,000 - £34,999 | 228 | 15% | 165 | 14% | 247 | 12% | 304 | 14% | 944 | 14% |  |  |
| £35,000-£60,000 | 288 | 18% | 259 | 22% | 422 | 20% | 420 | 20% | 1,389 | 20% |  |  |
| Over £60,000 | 250 | 16% | 205 | 17% | 307 | 15% | 356 | 17% | 1,118 | 16% |  |  |
| Prefer not to say/Did | 390 | 25% | 249 | 21% | 492 | 23% | 425 | 20% | 1,556 | 22% |  |  |
| Tenure |  |  |  |  |  |  |  |  |  |  |  |  |
| Own | 1,008 | 64% | 721 | 61% | 1,246 | 59% | 1,335 | 64% | 4,310 | 62% |  |  |
| Rent | 380 | 24% | 287 | 24% | 581 | 28% | 522 | 25% | 1,770 | 25% | 28.5435 | 0.001 |
| Live with friends or family | 121 | 8% | 135 | 11% | 220 | 10% | 167 | 8% | 643 | 9% |  |  |
| other | 32 | 2% | 20 | 2% | 35 | 2% | 37 | 2% | 124 | 2% |  |  |
| Missing | 27 | 2% | 16 | 1% | 23 | 1% | 39 | 2% | 105 | 2% |  |  |
| App installation |  |  |  |  |  |  |  |  |  |  |  |  |
| currently installed | 181 | 12% | 342 | 29% | 901 | 43% | 1,604 | 76% | 3,028 | 44% | 1.80E+03 | 0.000 |
| currently uninstalled | 313 | 20% | 288 | 24% | 406 | 19% | 238 | 11% | 1,245 | 18% |  |  |
| never installed | 1,074 | 68% | 549 | 47% | 798 | 38% | 258 | 12% | 2,679 | 39% |  |  |
| Trust |  |  |  |  |  |  |  |  |  |  |  |  |
| A great deal | 72 | 5% | 44 | 4% | 135 | 6% | 289 | 14% | 540 | 8% |  |  |
| A fair amount | 341 | 22% | 386 | 33% | 781 | 37% | 834 | 40% | 2,342 | 34% | 523.7157 | 0.000 |
| Not very much | 462 | 29% | 436 | 37% | 645 | 31% | 533 | 25% | 2,076 | 30% |  |  |
| Not at all | 630 | 40% | 288 | 24% | 419 | 20% | 393 | 19% | 1,730 | 25% |  |  |
| Don’t know | 63 | 4% | 25 | 2% | 125 | 6% | 51 | 2% | 264 | 4% |  |  |
| Ethnic |  |  |  |  |  |  |  |  |  |  |  |  |
| White | 1,408 | 23% | 1,051 | 17% | 1,837 | 30% | 1,874 | 30% | 6,170 | 100% | 7.0305 | 0.071 |
| Other | 160 | 20% | 128 | 16% | 268 | 34% | 226 | 29% | 782 | 100% |  |  |
| sex |  |  |  |  |  |  |  |  |  |  |  |  |
| Male | 856 | 26% | 504 | 15% | 889 | 27% | 1,040 | 32% | 3,289 | 100% | 69.0938 | 0.000 |
| Female | 712 | 19% | 675 | 18% | 1,216 | 33% | 1,060 | 29% | 3,663 | 100% |  |  |

# Table S13: WAVE 1 Post-estimation results of multinomial logistic regression model of factors associated with individuals belonging to different support subgroups for NHS COVID-19 app (Reference group – Completely supportive)

| **Variables** | **Class 1: Not supportive** | | | **Class 2: Ambivalent** | | | **Class 3: Somewhat supportive** | | |
| --- | --- | --- | --- | --- | --- | --- | --- | --- | --- |
|  | **RRR** | **95% CI** | **t** | **RRR** | **95% CI** | **t** | **RRR** | **95% CI** | **t** |
| Age | 1.00 | 0.99-1.02 | 0.22 | 1.00 | 0.99-1.02 | 0.68 | 0.99 | 0.98-1.06 | -1.11 |
| *Gender(Ref. Male)* | | | | | | | | | |
| Female | 1.31 | 0.93-1.86 | 1.53 | 1.54 | 1.17-2.03*** | 3.11 | 1.11 | 0.84-1.48 | 0.75 |
| *Ethnicity (Ref. All other ethnic groups)* | | | | | | | | | |
| White | 0.65 | 0.38-1.11 | -1.58 | 0.55 | 0.36-0.85*** | -2.71 | 0.79 | 0.50-1.25 | -0.99 |
| *Self-reported health status (Ref. Fair/bad/very bad)* | | | | | | | | | |
| Good/very good | 0.91 | 0.59-1.39 | -0.45 | 0.83 | 0.58-1.18 | -1.05 | 0.85 | 0.57-1.26 | -0.82 |
| *Disability or health problem lasting (or expected to last) at least 12 months (Ref. No)* | | | | | | | | | |
| Limited a lot/Limited a little | 1.09 | 0.7-1.68 | 0.37 | 1.00 | 0.69-1.45 | 0.02 | 0.95 | 0.64-1.41 | -0.26 |
| *Consider self-vulnerable to COVID-19 (Ref. No)* | | | | | | | | | |
| Yes | 1.24 | 0.81-1.89 | 0.99 | 0.90 | 0.65-1.24 | -0.66 | 1.06 | 0.76-1.48 | 0.34 |
| *Region (Ref. London and South)* | | | | | | | | | |
| North(Including Yorkshire) | 0.71 | 0.30-1.67 | -0.79 | 1.20 | 0.64-2.23 | 0.57 | 1.24 | 0.63-2.45 | 0.61 |
| Midlands and East of England | 0.82 | 0.35-1.90 | -0.47 | 1.21 | 0.66-2.22 | 0.61 | 1.26 | 0.64-2.47 | 0.66 |
| Wales | 0.75 | 0.33-1.74 | -0.66 | 1.14 | 0.62-2.10 | 0.43 | 1.34 | 0.69-2.62 | 0.87 |
| *Household Income category (Ref. Over £60,000)* | | | | | | | | | |
| Under £14,999 | 0.94 | 0.54-1.66 | -0.20 | 0.70 | 0.44-1.12 | -1.48 | 0.77 | 0.46-1.29 | -0.99 |
| £15,000 - £24,999 | 0.48 | 0.28-0.87*** | -2.66 | 0.56 | 0.37-0.86*** | -2.65 | 0.46 | 0.29-0.71*** | -3.49 |
| £25,000 - £34,999 | 0.94 | 0.55-1.61 | -0.22 | 0.83 | 0.54-1.28 | -0.85 | 0.73 | 0.46-1.15 | -1.36 |
| £35,000-£60,000 | 0.98 | 0.61-1.58 | -0.06 | 1.10 | 0.77-1.56 | 0.53 | 1.22 | 0.86-1.72 | 1.11 |
| *Housing ownership (Ref. other including living with friends/family)* | | | | | | | | | |
| Own | 0.44 | 0.25-0.78*** | -2.85 | 0.56 | 0.35-0.89** | -2.46 | 0.66 | 0.41-1.08 | -1.66 |
| Rent | 0.90 | 0.51-1.58 | -0.38 | 0.85 | 0.53-1.37 | -0.66 | 0.93 | 0.57-1.52 | -0.30 |
| *App installed at survey wave (Ref. Never installed)* | | | | | | | | | |
| Currently installed | 0.03 | 0.02-0.05*** | -15.47 | 0.09 | 0.06-0.12*** | -14.56 | 0.27 | 0.19-0.39*** | -6.99 |
| Currently uninstalled | 0.11 | 0.07-0.18*** | -8.74 | 0.19 | 0.13-0.29*** | -7.99 | 0.40 | 0.26-0.63*** | -4.02 |
| *Had or currently have COVID-19 since previous survey (Ref. Probably had it/Don’t know if had it*) | | | | | | | | | |
| Definitely had it | 0.82 | 0.17-4.06 | -0.24 | 1.06 | 0.33-3.35 | 0.09 | 1.38 | 0.48-3.98 | 0.60 |
| Haven’t had it | 0.76 | 0.54-1.07 | -1.56 | 0.86 | 0.65-1.13 | -1.09 | 0.94 | 0.70-1.25 | -0.43 |
| *Extent trust government to control the spread of COVID-19 (Ref. Not very much/Not all all/Don’t know)* | | | | | | | | | |
| A great deal/a fair amount | 0.19 | 0.12-0.29*** | -7.56 | 0.43 | 0.32-0.59*** | -5.38 | 0.57 | 0.42-0.77*** | -3.67 |
| *Extent concerned about the risk COVID-19 poses to self (Ref. Very concerned)* | | | | | | | | | |
| Fairly concerned | 1.14 | 0.70-1.85 | 0.53 | 1.35 | 0.9-1.96 | 1.58 | 2.11 | 1.42-3.14*** | 3.70 |
| Not very concerned | 2.11 | 1.17-3.79** | 2.50 | 1.59 | 1.00-2.53 | 1.95 | 2.26 | 1.39-3.70*** | 3.31 |
| Not at all concerned | 2.08 | 0.80-5.42 | 1.50 | 1.35 | 0.59-3.10 | 0.70 | 2.39 | 0.94-6.12 | 1.82 |
| *Extent concerned about the risk COVID-19 poses to the country (Ref. Very concerned)* | | | | | | | | | |
| Fairly concerned | 1.37 | 0.91-2.05 | 1.50 | 1.67 | 1.24-2.27*** | 3.34 | 1.36 | 1.00-1.86 | 1.94 |
| Not very concerned | 4.02 | 1.88-8.62*** | 3.59 | 2.36 | 1.186-4.71** | 2.45 | 0.99 | 0.42-2.31 | -0.03 |
| Not at all concerned | 10.87 | 2.63-44.85*** | 3.30 | 2.84 | 0.70-11.60 | 1.46 | 1.02 | 0.22-4.71 | 0.02 |
|  |  |  |  |  |  |  |  |  |  |
| Constant | 8.04 | 2.31-27.98*** | 3.28 | 6.72 | 2.52-17.94*** | 3.80 | 6.72 | 2.52-17.94*** | 3.80 |
| F | 6.30 |  |  |  |  |  |  |  |  |
| Prob>F | 0.000 |  |  |  |  |  |  |  |  |
| Number of observations | 1,978 |  |  |  |  |  |  |  |  |

Notes: RRR-Relative risk ratio. A RRR of less than 1 indicates that the risk of the outcome falling in the comparison group is *less* likely compared to the reference group. A RRR of more than 1 indicates that the risk of the outcome falling in the comparison group is *more* likely compared to the reference group; *** significant at 1% (p≤0.01); ** significant at 5%(p≤0.05); F= Fisher statistic. Prob= Probability. CI= Confidence interval.

# Table S14: WAVE 5 Post-estimation results of multinomial logistic regression model of factors associated with individuals belonging to different support subgroups for NHS COVID-19 app (Reference group –Supportive)

| **Variables** | **Class 1: Not supportive** | | | **Class 2: Ambivalent** | | |
| --- | --- | --- | --- | --- | --- | --- |
|  | **RRR** | **95% CI** | **t** | **RRR** | **95% CI** | **t** |
| Age | 1.01 | 0.99-1.02 | 0.95 | 0.99 | 0.98-1.00 | -1.91 |
| *Gender(Ref. Male)* | | | | | | |
| Female | 0.93 | 0.68-1.27 | -0.46 | 1.21 | 0.90-1.62 | 1.27 |
| *Ethnicity (Ref. All other ethnic groups)* | | | | | | |
| White | 0.70 | 0.43-1.16 | -1.37 | 0.70 | 0.44-1.10 | -1.56 |
| *Self-reported health status (Ref. Fair/bad/very bad)* | | | | | | |
| Good/very good | 1.27 | 0.85-1.89 | 1.18 | 1.15 | 0.78-1.70 | 0.7 |
| *Disability or health problem lasting (or expected to last) at least 12 months (Ref. No)* | | | | | | |
| Limited a lot/Limited a little | 0.85 | 0.55-1.29 | -0.78 | 0.95 | 0.63-1.43 | -0.25 |
| *Consider self-vulnerable to COVID-19 (Ref. No)* | | | | | | |
| Yes | 1.14 | 0.79-1.65 | 0.71 | 1.10 | 0.78-1.55 | 0.53 |
| *Region (Ref. London and South)* | | | | | | |
| North (including Yorkshire) | 0.73 | 0.36-1.48 | -0.87 | 1.13 | 0.59-2.16 | 0.36 |
| Midlands and East of England | 0.67 | 0.33-1.35 | -1.12 | 1.39 | 0.73-2.65 | 0.99 |
| Wales | 0.60 | 0.31-1.19 | -1.45 | 1.03 | 0.55-1.94 | 0.09 |
| *Household Income category (Ref. Over £60,000)* | | | | | | |
| Under £14,999 | 1.13 | 0.65-1.96 | 0.42 | 1.03 | 0.61-1.74 | 0.12 |
| £15,000 - £24,999 | 0.88 | 0.54-1.44 | -0.51 | 1.14 | 0.74-1.77 | 0.61 |
| £25,000 - £34,999 | 0.85 | 0.52-1.37 | -0.68 | 0.77 | 0.49-1.20 | -1.16 |
| £35,000-£60,000 | 1.05 | 0.71-1.56 | 0.26 | 0.91 | 0.62-1.33 | -0.49 |
| *Housing tenure (Ref. other including living with friends/family)* | | | | | | |
| Own | 1.13 | 0.67-1.91 | 0.44 | 1.25 | 0.77-2.03 | 0.89 |
| Rent | 1.07 | 0.62-1.84 | 0.24 | 1.30 | 0.79-2.14 | 1.02 |
| *App installation at current survey (Ref. Never installed)* | | | | | | |
| Currently installed | 0.07 | 0.06-0.10*** | -13.4 | 0.16 | 0.11-0.23*** | -9.38 |
| Currently uninstalled | 0.28 | 0.18-0.44*** | -5.55 | 0.41 | 0.26-0.64*** | -3.95 |
| *Had or currently have COVID-19 since previous survey (Probably had it/Don’t know)* | | | | | | |
| Definitely had it | 0.49 | 0.19-1.28 | -1.46 | 0.56 | 0.23-1.38 | -1.26 |
| Haven’t had it | 0.70 | 0.46-1.06 | -1.69 | 0.72 | 0.48-1.08 | -1.59 |
| *Extent trust government to control the spread of COVID-19 (not very much/not all /don’t know)* | | | | | | |
| Great deal/Fair amount | 0.27 | 0.20-0.38*** | -7.79 | 0.61 | 0.45-0.81*** | -3.35 |
| *Extent concerned about the risk COVID-19 poses to self (Ref. Very concerned)* | | | | | | |
| Fairly concerned | 1.44 | 0.88-2.36 | 1.46 | 0.91 | 0.59-1.41 | -0.43 |
| Not very concerned | 1.38 | 0.78-2.44 | 1.11 | 0.85 | 0.50-1.45 | -0.59 |
| Not at all concerned | 1.07 | 0.48-2.35 | 0.16 | 0.40 | 0.18-0.88** | -2.28 |
| *Extent concerned about the risk COVID-19 poses to the country (Ref. Very concerned)* | | | | | | |
| Fairly concerned | 1.41 | 0.96-2.08 | 1.76 | 1.56 | 1.08-2.24** | 2.38 |
| Not very concerned | 2.93 | 1.56-5.53*** | 3.33 | 3.03 | 1.60-5.73*** | 3.42 |
| Not at all concerned | 9.92 | 3.03-32.48*** | 3.79 | 2.29 | 0.52-10.08 | 1.09 |
|  |  |  |  |  |  |  |
| Constant | 7.26 | 2.40-22.00*** | 3.51 | 6.11 | 2.06-18.14*** | 3.26 |
| F | 6.27 |  |  |  |  |  |
| Prob>F | 0.000 |  |  |  |  |  |
| Number of observations | 1,562 |  |  |  |  |  |

Notes: RRR-Relative risk ratio. A RRR of less than 1 indicates that the risk of the outcome falling in the comparison group is *less* likely compared to the reference group. A RRR of more than 1 indicates that the risk of the outcome falling in the comparison group is *more* likely compared to the reference group; *** significant at 1% (p≤0.01); ** significant at 5%(p≤0.05). F= Fisher statistic. Prob= Probability. CI= Confidence interval.

# Table S15: WAVE 8 Post-estimation results of multinomial logistic regression model of factors associated with individuals belonging to different support subgroups for NHS COVID-19 app (Reference group – Supportive)

| Variables | **Class 1: Least supportive** | | | **Class 2: Less supportive** | | | **Class 3: Ambivalent** | | |
| --- | --- | --- | --- | --- | --- | --- | --- | --- | --- |
|  | **RRR** | **95% CI** | **t** | **RRR** | **95% CI** | **t** | **RRR** | **95% CI** | **t** |
| Age | 1.03 | 1.01-1.05*** | 2.72 | 1.00 | 0.98-1.01 | -0.47 | 1.00 | 0.99-1.02 | 0.28 |
| *Gender(Ref. Male)* | | | | | | | | | |
| Female | 1.24 | 0.81-1.89 | 0.98 | 1.59 | 1.01-2.50** | 2.00 | 1.28 | 0.89-1.83 | 1.33 |
| *Ethnicity (Ref. All other ethnic groups)* | | | | | | | | | |
| White | 0.54 | 0.27-1.11 | -1.68 | 0.60 | 0.29-1.22 | -1.42 | 0.75 | 0.42-1.34 | -0.97 |
| *Self-reported health status (Ref. Fair/bad/very bad)* | | | | | | | | | |
| Good/very good | 1.52 | 0.90-2.59 | 1.56 | 1.09 | 0.60-1.95 | 0.27 | 0.96 | 0.61-1.52 | -0.18 |
| *Disability or health problem lasting (or expected to last) at least 12 months (Ref. No)* | | | | | | | | | |
| Limited a lot/Limited a little | 1.52 | 0.91-2.57 | 1.59 | 1.04 | 0.57-1.89 | 0.13 | 1.04 | 0.65-1.66 | 0.15 |
| *Consider self-vulnerable to COVID-19 (Ref. No)* | | | | | | | | | |
| Yes | 0.97 | 0.57-1.67 | -0.1 | 0.92 | 0.54-1.56 | -0.32 | 0.80 | 0.53-1.22 | -1.04 |
| *Region (Ref. London and South)* | | | | | | | | | |
| North (including Yorkshire) | 1.09 | 0.47-2.51 | 0.19 | 0.82 | 0.32-2.05 | -0.43 | 1.24 | 0.56-2.71 | 0.53 |
| Midlands and East of England | 0.82 | 0.36-1.89 | -0.46 | 0.62 | 0.24-1.56 | -1.02 | 1.32 | 0.60-2.91 | 0.69 |
| Wales | 0.53 | 0.23-1.20 | -1.53 | 0.64 | 0.26-1.55 | -1 | 0.83 | 0.38-1.80 | -0.47 |
| *Household Income category (Ref. Over £60,000)* | | | | | | | | | |
| Under £14,999 | 0.67 | 0.31-1.42 | -1.05 | 0.59 | 0.28-1.28 | -1.33 | 0.75 | 0.41-1.39 | -0.91 |
| £15,000 - £24,999 | 0.89 | 0.45-1.79 | -0.32 | 0.91 | 0.45-1.83 | -0.27 | 1.38 | 0.81-2.36 | 1.18 |
| £25,000 - £34,999 | 0.76 | 0.41-1.43 | -0.84 | 0.48 | 0.24-0.97** | -2.04 | 0.68 | 0.39-1.17 | -1.41 |
| £35,000-£60,000 | 0.95 | 0.54-1.67 | -0.17 | 0.92 | 0.52-1.62 | -0.3 | 1.11 | 0.69-1.78 | 0.42 |
| *Housing tenure (Ref. other including living with friends/family)* | | | | | | | | | |
| Own | 0.67 | 0.28-1.57 | -0.92 | 0.81 | 0.35-1.87 | -0.5 | 0.61 | 0.31-1.19 | -1.44 |
| Rent | 0.68 | 0.28-1.65 | -0.85 | 0.87 | 0.37-2.06 | -0.31 | 0.95 | 0.48-1.88 | -0.15 |
| *App installation at current survey (Ref. Never installed)* | | | | | | | | | |
| Currently installed | 0.03 | 0.02-0.05*** | -13.1 | 0.09 | 0.05-0.16*** | -8.28 | 0.15 | 0.10-0.24*** | -8 |
| Currently uninstalled | 0.56 | 0.30-1.06 | -1.79 | 0.91 | 0.47-1.77 | -0.27 | 0.56 | 0.31-1.04 | -1.84 |
| *Had or currently have COVID-19 since previous survey (Probably had it/Don’t know)* | | | | | | | | | |
| Definitely had it | 1.09 | 0.42-2.85 | 0.18 | 2.61 | 0.98-6.93 | 1.92 | 0.51 | 0.20-1.30 | -1.41 |
| Haven’t had it | 0.75 | 0.42-1.37 | -0.93 | 1.83 | 0.89-3.79 | 1.64 | 1.20 | 0.71-2.05 | 0.68 |
| *Extent trust government to control the spread of COVID-19 (not very much/not all /don’t know)* | | | | | | | | | |
| Great deal/Fair amount | 0.30 | 0.19-0.47*** | -5.09 | 0.49 | 0.31-0.78*** | -3.04 | 0.57 | 0.39-0.83*** | -2.97 |
| *Extent concerned about the risk COVID-19 poses to self (Ref. Very concerned)* | | | | | | | | | |
| Fairly concerned | 0.82 | 0.41-1.64 | -0.57 | 2.26 | 1.00-5.07** | 1.97 | 1.04 | 0.61-1.79 | 0.14 |
| Not very concerned | 1.31 | 0.56-3.03 | 0.62 | 3.36 | 1.31-8.70** | 2.52 | 1.56 | 0.8-3.10 | 1.28 |
| Not at all concerned | 2.05 | 0.56-7.45 | 1.09 | 4.72 | 1.07-20.76** | 2.05 | 1.08 | 0.30-3.8 | 0.11 |
| *Extent concerned about the risk COVID-19 poses to the country (Ref. Very concerned)* | | | | | | | | | |
| Fairly concerned | 1.40 | 0.75-2.64 | 1.05 | 2.18 | 1.11-4.29** | 2.25 | 1.19 | 0.75-1.90 | 0.73 |
| Not very concerned | 5.76 | 2.25-14.79*** | 3.65 | 5.09 | 1.85-14.03*** | 3.15 | 1.81 | 0.78-4.20 | 1.38 |
| Not at all concerned | 13.72 | 2.56-73.62*** | 3.06 | 3.13 | 0.41-24.01 | 1.1 | 1.93 | 0.31-12.11 | 0.7 |
|  |  |  |  |  |  |  |  |  |  |
| Constant | 2.87 | 0.62-13.35 | 1.34 | 0.97 | 0.19-5.12 | -0.03** | 4.93 | 1.31-18.60 | 2.36 |
| F | 5.13 |  |  |  |  |  |  |  |  |
| Prob>F | 0.000 |  |  |  |  |  |  |  |  |
| Number of observations | 1,194 |  |  |  |  |  |  |  |  |

Notes: RRR-Relative risk ratio. A RRR of less than 1 indicates that the risk of the outcome falling in the comparison group is *less* likely compared to the reference group. A RRR of more than 1 indicates that the risk of the outcome falling in the comparison group is *more* likely compared to the reference group; *** significant at 1% (p≤0.01); ** significant at 5%(p≤0.05). F= Fisher statistic. Prob= Probability. CI= Confidence interval.
