# Supplementary material for: Characteristics Influencing Support for the National Health Service COVID-19 App in England and Wales: Findings From a Longitudinal Survey
Source: J Med Internet Res. 2026 Jan 28;28:e76863. doi: 10.2196/76863 (PMC12895152; doi:10.2196/76863)
Supplement: Multimedia Appendix 5 [file jmir_v28i1e76863_app5.docx]

**Table S1.** Summary of multinomial logistic regression model highlighting those factors that were positively (green) or negatively (orange) associated with subgroup membership compared to the most supportive group at survey waves 1, 5 and 8.

| **Variables** | **Wave 1** | | | **Wave 5** | | **Wave 8** | | |
| --- | --- | --- | --- | --- | --- | --- | --- | --- |
|  | **Class 1: Not supportive** | **Class 2: Ambivalent** | **Class 3: Somewhat supportive** | **Class 1: Not supportive** | **Class 2: Ambivalent** | **Class 1: Least supportive** | **Class 2: Less supportive** | **Class 2: Ambivalent** |
| Age |  |  |  |  |  | *** |  |  |
| *Gender(Ref. Male)* | | | | | | | | |
| Female |  | *** |  |  |  |  | ** |  |
| *Ethnicity (Ref. All other ethnic groups)* | | | | | | | | |
| White |  | *** |  |  |  |  |  |  |
| *Self-reported health status (Ref. Fair/bad/very bad)* | | | | | | | | |
| Good/very good |  |  |  |  |  |  |  |  |
| *Disability or health problem lasting (or expected to last) at least 12 months (Ref. No)* | | | |  | |  | | |
| Limited a lot/Limited a little |  |  |  |  |  |  |  |  |
| *Consider self-vulnerable to COVID-19 (Ref. No)* | | | | | | | | |
| Yes |  |  |  |  |  |  |  |  |
| *Region (Ref. London and South)* | | | | | | | | |
| North(Including Yorkshire) |  |  |  |  |  |  |  |  |
| Midlands and East of England |  |  |  |  |  |  |  |  |
| Wales |  |  |  |  |  |  |  |  |
| *Household Income category (Ref. Over £60,000)* | | | | | | | | |
| Under £14,999 |  |  |  |  |  |  |  |  |
| £15,000 - £24,999 | *** | *** | *** |  |  |  |  |  |
| £25,000 - £34,999 |  |  |  |  |  |  | ** |  |
| £35,000-£60,000 |  |  |  |  |  |  |  |  |
| *Housing ownership (Ref. other including living with friends/family)* | | | | | | | | |
| Own | *** | ** |  |  |  |  |  |  |
| Rent |  |  |  |  |  |  |  |  |
| *App installed at survey wave (Ref. Never installed)* | | | | | | | | |
| Currently installed | *** | *** | *** | *** | *** | *** | *** | *** |
| Currently uninstalled | *** | *** | *** | *** | *** |  |  |  |
| *Had or currently have COVID-19 since previous survey (Ref. Probably had it/Don’t know if had it*) | | | | | | | | |
| Definitely had it |  |  |  |  |  |  |  |  |
| Haven’t had it |  |  |  |  |  |  |  |  |
| *Extent trust government to control the spread of COVID-19 (Ref. Not very much/Not all all/Don’t know)* | | | | | | | | |
| A great deal/a fair amount | *** | *** | *** | *** | *** | *** | *** | *** |
| *Extent concerned about the risk COVID-19 poses to self (Ref. Very concerned)* | | | | | | | | |
| Fairly concerned |  |  | *** |  |  |  | ** |  |
| Not very concerned | ** |  | *** |  |  |  | ** |  |
| Not at all concerned |  |  |  |  | ** |  | ** |  |
| *Extent concerned about the risk COVID-19 poses to the country (Ref. Very concerned)* | | | | | | | | |
| Fairly concerned |  | *** |  |  | ** |  | ** |  |
| Not very concerned | *** | ** |  | *** | *** | *** | *** |  |
| Not at all concerned | *** |  |  | *** |  | *** |  |  |

Notes: Orange indicates that the risk of the outcome falling in the comparison group is *less* likely compared to the reference group. Green indicates that the risk of the outcome falling in the comparison group is *more* likely compared to the reference group; *** significant at 1% (p≤0.01); ** significant at 5%(p≤0.05). Blank indicates no significant difference
